# Rising global burden of common gynecological diseases in women of childbearing age from 1990 to 2021: an update from the Global Burden of Disease Study 2021

**DOI:** 10.1186/s12978-025-02013-1

**Published:** 2025-04-21

**Authors:** Yidan Gao, Xuemei Wang, Qian Wang, Lijuan Jiang, Cuixiu Wu, Yuanshuo Guo, Na Cui, Haoneng Tang, Lingli Tang

**Affiliations:** Department of Laboratory Medicine, The Second Xiangya Hospital of Central South University, Changsha, 410011 Hunan China

**Keywords:** Gynecological diseases, Global burden of disease, Epidemiology

## Abstract

**Background:**

Gynecological diseases significantly impact the reproductive health in women of childbearing age (WCBA). However, there is currently a lack of analysis on the specific burden and forecast of common gynecological diseases for this population. The aim of this study was to provide new details for gynecological disorders in WCBA from 1990 to 2021 worldwide based on the Global Burden of Disease (GBD) data.

**Methods:**

Employing data from the GBD 2021, we analyzed the disability-adjusted life years (DALYs), death, incidence, and prevalence for thirteen types of gynecological disorders by socioeconomic development index (SDI), age, year, and location in WCBA. Age-standardized rates were used to compare the burdens across various time periods and locations. Temporal trends were assessed using Joinpoint regression analysis from 1990 to 2021, the Bayesian age-period-cohort (BAPC) model projected the disease burden through 2031, and the Nordpred model conducted a sensitivity analysis of the prediction results, which validated the findings' reliability.

**Results:**

From 1990 to 2021, the age–standardized DALYs rate (ASDR) and mortality rate (ASMR) of combined gynecological diseases (CGDs) increased by AAPC of 0.28% (95% CI 0.19 to 0.36) and 0.53% (95% CI 0.25 to 0.82) among WCBA. Furthermore, the projections showed a declining trend in the next decade, with ASDR and ASMR dropping by 11.06% and 25.23%, respectively. Notably, HIV/AIDS, polycystic ovary syndrome (PCOS), infertility, premenstrual syndrome (PMS) were the key drivers behind the increased ASDR of CGDs. In 2021, HIV/AIDS (16.38 million), PMS (7.43 million), and cervical cancer (4.18 million) topped the list for the absolute number of DALYs globally among WCBA. CGDs burdens were negatively correlated with and SDI. Women aged 15–24 years showed the most significant rise in CGDs burdens, highlighting its increasingly affecting younger women. The DALYs and death of malignant gynecological tumors are most significant in women aged 40–49 years, the incidence of PCOS predominantly occurs in 15–19 years, and infertility prevalence peaks at 30–39 years.

**Conclusions:**

The global CGDs burden has increased over the last 32 years, and higher in lower SDI countries. Immediate and effective interventions should be taken to target different types of gynecological disorders, age groups, and countries with high gynecological disease burdens. These findings would provide targeted insights for public health policies and interventions enhancing reproductive health in women of childbearing age.

**Supplementary Information:**

The online version contains supplementary material available at 10.1186/s12978-025-02013-1.

## Abstract (plain language summary)

This comprehensive analysis spanning from 1990 to 2021 reveals a significant rise in age-standardized disability-adjusted life years (DALYs) and mortality rates for combined gynecological diseases (CGDs) in women of childbearing age (WCBA).

**Supplementary Information:**

The online version contains supplementary material available at 10.1186/s12978-025-02013-1.

## Background

Gynecological disorders, characterized by insidious onset and high recurrence, predominantly affect women of childbearing age [1], causing chronic pain [2], reproductive dysfunction [3], psychological problems and poor pregnancy outcomes [4]. This study focused on women of childbearing age, who facing higher risks of gynecological diseases due to their physiological characteristics and reproductive behaviors.

Gynecological disorders include but not limited to reproductive tract infections, menstrual disorders, endocrine-related conditions, benign and malignant tumors, and infertility. For comprehensive disease burden assessment, combined gynecological disorders (CGDs) are defined in this study as a composite of 13 conditions extracted from the Global Burden of Disease (GBD) 2021 database: sexually transmitted infections (HIV/AIDS and others), premenstrual syndrome (PMS), endometriosis, female infertility, polycystic ovarian syndrome (PCOS), uterine fibroids, genital prolapse, cervical cancer, uterine cancer, ovarian cancer, benign and in situ cervical and uterine neoplasms, and other gynecological diseases.

Despite global attention on women's health, a 2022 report from the World Health Organization (WHO) indicates that progress towards achieving women's health goals remains slow [5]. The COVID-19 pandemic has exacerbated existing gender inequalities, presenting new health challenges for women [6]. Furthermore, the WHO's Sustainable Development Goal 3 aims to significantly reduce global maternal mortality rates and improve reproductive health by 2030 [7]. Previous epidemiological studies on gynecological diseases have primarily focused on individual conditions and relied on outdated data. For instance, earlier analyses concentrated specifically on female cancers such as cervical, ovarian, and uterine cancers, as well as conditions like PCOS and premenstrual syndrome [8–10], failing to holistically evaluate the comprehensive burden of gynecological disorders. Additionally, most studies utilized data up to 2019 [11], not reflecting healthcare trends during the COVID-19 pandemic. Particularly in underdeveloped regions with limited healthcare resource, the burden of gynecological diseases remains unclear [12, 13]. These gaps underscore the need for a comprehensive assessment of the burdens associated with gynecological diseases to better inform policy interventions.

The Global Burden of Diseases, Injuries, and Risk Factors Study (GBD) 2021 provides a comprehensive dataset for epidemiological research, extensively applied in disease burden and health inequalities [14, 15]. In our study, we leverage the latest GBD 2021 data to estimate the burden of gynecological disorders among WCBA by Socio-demographic Index (SDI) and age across global, regional, and national levels from 1990 to 2021, predicting its changing trend for the next decade, to provide a reference for decision–makers.

## Methods

### Study population and data collection

GBD 2021 dataset assesses health loss due to 371 diseases and 88 risk factors across 204 countries since 1990, employing advanced methodologies like spatiotemporal Gaussian process regression and MR-BRT to correct biases and estimate health loss [16, 17]. The more details of methodologies for estimating gynecological diseases burden in GBD 2021 is described in previous studies [16]. To assess the latest burden of female cancers (cervical, uterine, and ovarian cancer) in 2022, we retrieved data from the International Agency for Research on Cancer’s GLOBOCAN 2022 estimates available online at Cancer Today [18] (https://gco.iarc.who.int/today/en/dataviz/tables).

The list of International Classification of Diseases (ICD) 10 codes for each type of gynecological disorders included in this study are available in Appendix 1. This list is not exhaustive as some gynecological conditions, like menstruation-related issues, couldn't be estimated in GBD. However, it is the most comprehensive analysis of gynecological health loss among childbearing women to date.

Women aged 15–49 years were included in this study based on the definition of women of childbearing age from the WHO [19]. The estimates and 95% UIs for the annual incidence, prevalence, death and the disability-adjusted life years (DALYs) of gynecological disorders across 7 age groups (15–19, 20–24, 25–29, 30–34, 35–39, 40–44, 45–49 years) spanning 1990 to 2021 were extracted from GBD 2021 Results [20] (http://ghdx.healthdata.org/gbd-results-tool) (accessed on May 22nd, 2024) at global, regional and national levels. We also utilized the Socio-demographic index (SDI) as a key metric to assess burden disparities across regions. The SDI is a composite indicator of the social and economic conditions influencing health outcomes, integrating total fertility rate under the age of 25 (TFU25), mean years of education for the population aged 15 and older (EDU15 +), and lag-distributed income (LDI) per capita. Spanning from 0 to 1, it classifies countries into five tiers: low SDI, low-middle SDI, middle SDI, high-middle SDI, and high SDI [21]. We applied Pearson's correlation coefficient to assess the association between burden and SDI.

### Statistics analysis

To compare burden of populations with different age structures or within the same population over time, we calculated the age-standardized rates (ASR) with corresponding 95% confidence intervals (CIs) in WCBA by direct method, based on the world standard population reported in the Global Burden of Disease Study 2021 [22].

The ASR (per 100,000 population), which accounts for incidence, prevalence, mortality, and DALYs, is determined by the formula:

$${\text{ASR }} = { }\frac{{\mathop \sum \nolimits_{{{\text{i}} = 1}}^{{\text{A}}} {\text{a}}_{{\text{i}}} {\text{w}}_{{\text{i}}} }}{{\mathop \sum \nolimits_{{{\text{i}} = 1}}^{{\text{A}}} {\text{w}}_{{\text{i}}} }}{ } \times { }100,000$$

where ${\text{a}}_{\text{i}}$ is the age-specific rate for age group $\text{i}$, and ${\text{w}}_{\text{i}}$ is the weight of the age group in the standard population and A denotes the number of age groups [23].

We utilized the Joinpoint Regression program (version 5.2.0) to analyze trends in gynecological diseases from 1990 to 2021. This analysis identified significant changes in the time-series data. The joinpoint regression model calculates the annual percentage change (APC) and 95% confidence intervals (CIs) for each period, with the average annual percentage change (AAPC) as a weighted average of APCs on segmented intervals [24]. Significance testing is conducted by Monte Carlo Permutation approach. The analytical models can account for the estimated variability of each data point or employ a Poisson model to represent the variability [25].

The formula of AAPC as follows:

$${\text{AAPC}} = \left\{ {{\text{exp}}\left( {\frac{{\sum {\text{ w}}_{{\text{i}}} {\text{b}}_{{\text{i}}} }}{{\sum {\text{ w}}_{{\text{i}}} }}} \right) - 1} \right\} \times 100$$

in which ${\text{w}}_{\text{i}}$ indicates the length of each segment in the range of years, while the ${\text{b}}_{\text{i}}$ is the slope coefficient for each segment in the desired range of years [23].

An increasing trend is indicated by both AAPC and its 95% CI lower bound above zero, while a decreasing trend is shown by both AAPC and its upper boundary of 95% CI below zero; a stable trend is indicated if the 95% CI includes zero.

To predict the future burden of gynecological diseases from 2022 to 2031, we used the Bayesian Age-Period-Cohort (BAPC) model integrating nested laplace approximations, implemented by the BAPC package. This model facilitates the approximation of marginal posterior distributions, effectively avoiding some of the mixing and convergence issues associated with the Markov Chain Monte Carlo sampling technique traditionally employed in Bayesian methodologies [26]. To assess the robustness of the predicted results, we used Nordpred package for analysis. The global population forecast information for 2017–2100 was obtained from GBD study [27].

Visualizations were created using the ggplot2 package. All data cleaning, statistical analyses and visualization were conducted using R software (version 4.3.3), with a *P*-value < 0.05 considered statistically significant.

## Results

### Global burden of gynecological diseases in WCBA

**Fig. 1:**
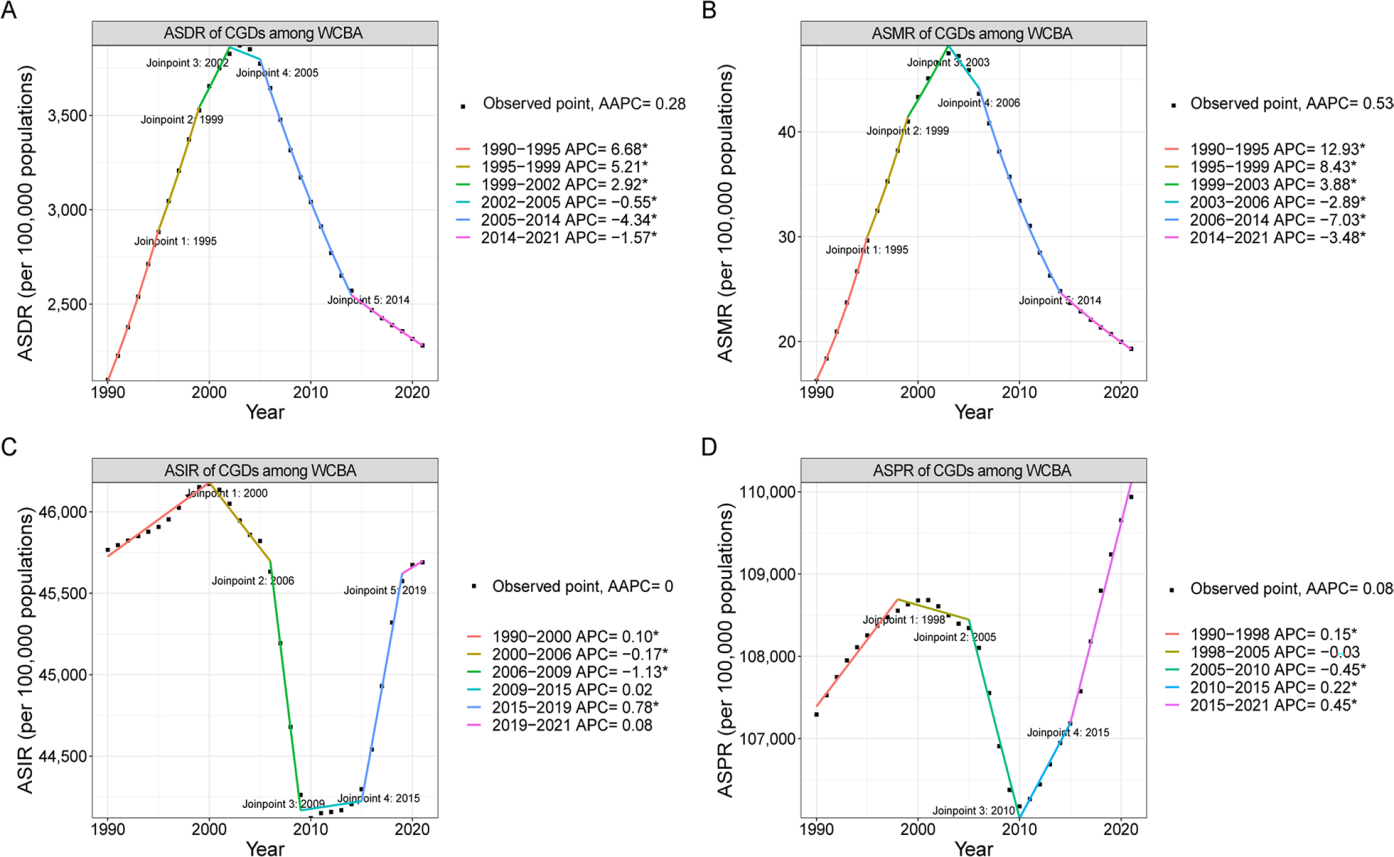
Global burden trends of combined gynecological disorders (CGDs) in women of childbearing age from 1990 to 2021. **A** Age-standardized DALYs rate; **B** Age-standardized mortality rate; **C** Age-standardized incidence rate; **D** Age-standardized prevalence rate. * Indicates that the Annual Percent Change (APC) is significantly different from zero at the alpha = 0.05 level. *AAPC* Average annual percentage change, *DALYs* Disability-adjusted life-years

In 2021, 899.07 million new cases and 2170.14 million prevalent cases of combined gynecological diseases (CGDs) in women of childbearing age (WCBA) were documented, with 0.39 million death cases (Table 1), which comprised 12.34% of global all cause death in WCBA. There were 45.22 million DALYs recorded in the same year (Table 1). Over the past 32 years, the global age-standardized DALYs and mortality rates (ASDR and ASMR) of CGDs in WCBA have increased by AAPC of 0.28% (95% CI 0.19% to 0.36%) and 0.53% (0.25% to 0.82%), respectively (Table 1). Specifically, from 1990 to 2003, ASDR and ASMR notably increased, but declined consistently over the subsequent 18 years (Fig. 1). The age-standardized prevalence rate (ASPR) increased with AAPC of 0.08% (0.06% to 0.1%), whereas the age-standardized incidence rate (ASIR) was approximately stable (AAPC, 0% [− 0.02% to 0.02%]).

**Table 1:** Burden of combined gynecological diseases (CGDs) among women of childbearing age in 2021 and their average percentage changes from 1990 to 2021 at the global and regional levels

Characteristics	DALYs (95% CI)				Death (95% CI)				Incidence (95% CI)				Prevalence (95% CI)			
	Count	ASDR (per 100,000 population)	AAPC for ASDR (%)	*P*-value	Count	ASMR (per 100,000 population)	AAPC for ASMR (%)	*P*-value	Count	ASIR (per 100,000 population)	AAPC for ASIR (%)	*P*-value	Count	ASPR (per 100,000 population)	AAPC for ASPR (%)	*P*-value
Global	45,218,531.35 (31,315,646.01–64911062.18)	2280.68 (1577.01–3278.47)	0.28 (0.19 to 0.36)	< 0.001	385,713.81 (310,300.91–483,620.37)	19.3 (15.51–24.22)	0.53 (0.25 to 0.82)	< 0.001	899,068,453.31 (611,860,823.87–1,232,400,408.47)	45,690.02 (31,094.86–62,601.32)	0 (− 0.02 to 0.02)	0.849	2,170,136,352.21 (1,564,197,137.89–2,913,885,604.92)	109,937.36 (79,173.46–147,739.67)	0.08 (0.06 to 0.1)	< 0.001
Age group (years)																
15–19	2,250,171.59 (1,436,090.38–3,480,124.99)	741.04 (472.94–1146.1)	0.52 (0.42 to 0.61)	< 0.001	14,274.23 (10,275.99–19,152.4)	4.7 (3.38–6.31)	1.2 (0.89 to 1.52)	< 0.001	69,099,273.87 (43,516,689.89–97,723,279.28)	22,756.23 (14,331.2–32,182.87)	0.01 (− 0.01 to 0.03)	0.203	150,366,384.34 (105,876,194.62–211,456,574.76)	49,519.64 (34,867.84–69,638.27)	0.06 (0.05 to 0.08)	< 0.001
20–24	4,326,617.14 (2,840,789.37–6,542,927.4)	1472.88 (967.07–2227.36)	0.21 (0.07 to 0.34)	0.002	27,630.55 (21,118.04–35984.06)	9.41 (7.19–12.25)	0.32 (− 0.26 to 0.89)	0.281	105,234,104.72 (73,445,639.99–140,171,224.61)	35,824.13 (25,002.6–47,717.53)	0.13 (0.11 to 0.16)	< 0.001	246,092,908.39 (171,369,722.5–332,465,053.82)	83,775.73 (58,338.23–113,178.81)	0.25 (0.24 to 0.27)	< 0.001
25–29	6,597,050.7 (4,359,341.24–9,809,073.28)	2267.13 (1498.12–3370.96)	0.12 (− 0.1 to 0.35)	0.268	44,444.99 (33,934.77–58,249.06)	15.27 (11.66–20.02)	0.25 (− 0.28 to 0.78)	0.362	145,094,161.15 (99,436,606.34–199,314,020.42)	49,862.72 (34,172.15–68,495.79)	0.08 (0.02 to 0.14)	0.007	313,619,092.96 (224,768,552.82–429,325,332.63)	107,777.6 (77,243.43–147,540.93)	0.17 (0.16 to 0.18)	< 0.001
30–34	8,370,434.09 (5,769,371.57–11,923,658.25)	2800.12 (1930–3988.77)	0.3 (0.04 to 0.56)	0.025	63,838.84 (50,167.44–81,390.64)	21.36 (16.78–27.23)	0.68 (0.38 to 0.98)	< 0.001	174,689,165.28 (125,180,319.74–231,452,331.13)	58,437.98 (41,876.01–77426.71)	0.01 (− 0.03 to 0.04)	0.676	379,850,007.46 (266,986,996.03–525614475.27)	127,069.51 (89,313.96–175,831.45)	0.07 (0.05 to 0.1)	< 0.001
35–39	8,615,690.61 (6,085,003.27–12,192,294.64)	3101.37 (2190.4–4388.83)	0.51 (0.33 to 0.69)	< 0.001	71,620.24 (58,685.44–89,111.2)	25.78 (21.12–32.08)	1.08 (0.59 to 1.58)	< 0.001	171,726,382.4 (116,118,654.14–230,737,885.63)	61,815.94 (41,798.96–83,058.18)	0 (− 0.02 to 0.01)	0.576	413,172,476.07 (299,257,436.62–551,708,844.34)	148,728.73 (107,723–198597.34)	0.07 (0.04 to 0.11)	< 0.001
40–44	8,129,470.94 (5,780,579.29–11,396,064.44)	3276.83 (2330.03–4593.52)	0.4 (0.25 to 0.54)	< 0.001	80,079.8 (66,788.4–95,370.08)	32.28 (26.92–38.44)	0.75 (0.45 to 1.06)	< 0.001	137,754,776.18 (93,930,621.77–189,083,792.73)	55,526.16 (37,861.53–76,215.85)	− 0.07 (− 0.1 to − 0.04)	< 0.001	378,506,880.6 (276,185,538.05–495340839.15)	152,568.46 (111,324.8–199,661.86)	0.03 (0.02 to 0.05)	< 0.001
45–49	6,929,096.29 (5,044,470.9–9,566,919.19)	2940.51 (2140.73–4059.93)	− 0.07 (− 0.12 to − 0.01)	0.025	83,825.15 (69,330.84–104,362.94)	35.57 (29.42–44.29)	0.02 (− 0.16 to 0.2)	0.846	95,470,589.71 (60,232,292–143917874.66)	40,515 (25,560.87–61,074.65)	− 0.22 (− 0.28 to − 0.16)	< 0.001	288,528,602.4 (219,752,697.25–367,974,484.94)	122,443.32 (93,256.79–156,157.89)	-0.1 (-0.11 to -0.09)	< 0.001
Socio-demographic index																
High SDI	3,400,377.04 (2,125,400.56–5,239,395.27)	1304.84 (806.64–2027.67)	− 0.44 (− 0.54 to − 0.34)	< 0.001	10,245.34 (9795.94–10,716.84)	3.54 (3.38–3.7)	− 2 (− 2.11 to − 1.89)	< 0.001	100,675,641.66 (67,567,891.53–140,495,159.03)	39,916.14 (26,792.84–55,619.06)	− 0.04 (− 0.07 to − 0.02)	< 0.001	256,974,323.16 (182,941,017.34–348,912,628.87)	100,274.97 (71,147.26–136,663.45)	0 (-0.02 to 0.02)	0.918
High-middle SDI	5,024,101.42 (3,283,940.19–7,538,314.14)	1491.65 (963.22–2262.36)	− 0.12 (− 0.22 to − 0.01)	0.034	26,426.15 (23,645.77–30,003.61)	7.19 (6.44–8.16)	0.14 (− 0.14 to 0.42)	0.32	136,305,720.5 (91,873,799.35–189,488,971.87)	42,585.68 (28,698.11–59,128.32)	− 0.2 (− 0.24 to − 0.16)	< 0.001	335,086,121.82 (236,162,461.49–458,842,178.38)	102,868.5 (72,166.86–141,421.41)	-0.07 (-0.09 to -0.06)	< 0.001
Middle SDI	12,956,237.56 (9,327,570.27–18,097,545.78)	2014.03 (1445.99–2822.94)	0.72 (0.46 to 0.98)	< 0.001	103,229.67 (91,927.2–115,809.35)	15.73 (14.01–17.66)	1.66 (1.24 to 2.08)	< 0.001	281,655,731.62 (190,949,160.13–389,184,350.22)	44,565.13 (30,201.05–61512.42)	− 0.1 (− 0.21 to 0)	0.052	693,863,497.59 (498,965,267.75–934,896,753.18)	109,074.61 (78,258.1–147,227.05)	0.07 (0.05 to 0.09)	< 0.001
Low-middle SDI	13,151,292.13 (8,743,110.34–19,251,559.74)	2673.86 (1785.08–3902.72)	0.41 (0.22 to 0.6)	< 0.001	126,855.92 (92,391.67–171,251)	26.23 (19.19–35.31)	0.89 (0.49 to 1.28)	< 0.001	231,047,085.72 (157,169,099.95–316,234,957.23)	46,192.24 (31,395.81–63,298.86)	0.04 (0.02 to 0.06)	0.001	559,799,991.53 (402,788,112.31–751,419,968.94)	112,650.6 (81,206.32–150,935.99)	0.09 (0.08 to 0.1)	< 0.001
Low SDI	10,651,764.35 (7,341,209.58–15,256,560.26)	4309.35 (2976.45–6162.53)	− 1.26 (− 1.72 to − 0.8)	< 0.001	118,640.82 (85,532.49–164,509.73)	49.54 (35.71–68.85)	− 1.71 (− 2.37 to − 1.04)	< 0.001	148,645,224.91 (101,778,909.34–202,178,534.34)	57,770.87 (39,512.94–78,726.34)	0.01 (− 0.01 to 0.03)	0.226	322,718,686.03 (238,316,626.41–422,988,455.14)	127,041.23 (94,254.24–165,561.91)	0.01 (-0.01 to 0.03)	0.432
Region																
Andean Latin America	337,502.69 (214,609.92–515,104.65)	1926.77 (1225.74–2940.26)	− 0.17 (− 0.34 to 0.01)	0.061	2051.48 (1576.78–2703.03)	11.84 (9.09–15.61)	− 0.61 (− 1.41 to 0.19)	0.133	8,927,722.36 (5,998,537.14–12,396,606.29)	50,768.04 (34,069.29–70,543.95)	0 (− 0.03 to 0.03)	0.9	23,706,471.05 (17,556,784.32–31,251,295.52)	135,206.6 (100,133.64–178,220.73)	0.05 (0.02 to 0.07)	< 0.001
Australasia	98,368.5 (57,424.33–158,233.63)	1289.13 (748.11–2085.39)	− 0.49 (− 0.6 to − 0.38)	< 0.001	196.87 (161.26–239.14)	2.37 (1.94–2.88)	− 2.77 (− 3.58 to − 1.95)	< 0.001	2,816,971.11 (1,858,714.35–3,979,023.9)	37,821.02 (24,942.83–53,438.01)	− 0.11 (− 0.13 to − 0.09)	< 0.001	7,189,951.29 (5,140,752.37–9,720,935.94)	95,791.04 (68,279.72–129,876.7)	-0.21 (-0.24 to -0.18)	< 0.001
Caribbean	355,191.04 (220,855.18–546,964.5)	2907.82 (1804.71–4483.03)	− 0.64 (− 1.05 to − 0.23)	0.002	3983.69 (2600.38–5926.73)	32.43 (21.12–48.3)	− 0.91 (− 1.52 to − 0.29)	0.004	6,247,324.93 (4,148,830.17–8,736,784.65)	51,540.17 (34,232.69–72,045.36)	− 0.01 (− 0.02 to 0)	0.006	15,135,169.24 (11,043,304.01–20001195)	124,576.98 (90,839.54–164,737.54)	-0.02 (-0.02 to -0.01)	< 0.001
Central Asia	366,791.63 (235,461.62–555,599.78)	1456.64 (933.83–2210.54)	− 0.16 (− 0.25 to − 0.06)	0.001	1877.56 (1644.2–2144.75)	7.45 (6.52–8.51)	− 0.46 (− 0.88 to − 0.05)	0.028	13,722,610.63 (9,223,995.94–19,257,560.05)	55,127.44 (36,978.26–77,426.89)	− 0.04 (− 0.06 to − 0.01)	0.003	26,388,168.03 (18,721,001.21–35,945,389.3)	105,985.43 (75,152.35–144,290.65)	0 (-0.01 to 0.01)	0.952
Central Europe	311,253.03 (201,474.03–470764.54)	1085.31 (687.15–1669.69)	− 0.78 (− 0.87 to − 0.7)	< 0.001	1834.86 (1626.89–2055.16)	5.43 (4.81–6.07)	− 2.17 (− 2.42 to − 1.93)	< 0.001	9,631,347.32 (6,417,973.2–13,578,875.24)	36,577.31 (24,433.57–51,374.03)	− 0.14 (− 0.17 to − 0.12)	< 0.001	23,532,650.79 (16,434,475.64–32,502,739.08)	86,287.91 (59,810.32–119,881.01)	-0.05 (-0.07 to -0.03)	< 0.001
Central Latin America	1,101,660.88 (743,886.26–1,630,749.53)	1597.28 (1077.37–2367.16)	− 0.41 (− 0.51 to − 0.31)	< 0.001	7935.48 (6999.91–8907.97)	11.4 (10.06–12.8)	− 0.73 (− 0.85 to − 0.62)	< 0.001	36,025,432.68 (24,022,314.14–50,716,171.38)	52,561.24 (35,053.44–73,932.57)	− 0.12 (− 0.17 to − 0.07)	< 0.001	85,621,819.59 (63,000,352.57–112,980,253.2)	124,588.54 (91,610.41–164,518.95)	-0.04 (-0.06 to -0.01)	0.003
Central Sub-Saharan Africa	1,710,397.81 (1,086,121.17–2,554,730.87)	5933.85 (3809.54–8771.11)	− 0.7 (− 1.26 to − 0.14)	0.014	21,241.14 (13,534.7–31,455.12)	76.2 (49.13–111.61)	− 0.85 (− 1.42 to − 0.29)	0.003	18,485,195.1 (12,755,561.03–24956819.73)	60,800.48 (41,939.97–82,000.75)	− 0.02 (− 0.03 to − 0.01)	0.001	46,618,649.93 (35,002,612.73–60,304,671.45)	154,947.14 (117,015.14–198,973.52)	0.01 (-0.01 to 0.03)	0.185
East Asia	4,256,516.56 (2,482,516.15–6,854,585.55)	1168.13 (675.55–1896.64)	− 0.57 (− 0.62 to − 0.52)	< 0.001	18,965.47 (13,544.72–25,385.95)	4.61 (3.29–6.15)	− 0.7 (− 0.92 to − 0.48)	< 0.001	131,561,347.19 (87,298,889.56–187,114,024.63)	37,966.8 (25,144.05–53866.55)	− 0.35 (− 0.59 to − 0.11)	0.005	328,184,010.63 (224,603,821.6–462,536,467.61)	93,067.85 (63,444.11–131,468.74)	-0.14 (-0.24 to -0.05)	0.004
Eastern Europe	1,344,909.48 (1,000,022.45–1,839,022.38)	2409.28 (1771.71–3333.28)	1.06 (0.81 to 1.31)	< 0.001	11,924.13 (11,308.98–12,617.2)	20.08 (19.1–21.19)	2.68 (2.1 to 3.27)	< 0.001	26,984,105.81 (17,988,009.41–37,707,743.14)	51,988.93 (34,636.32–72,769.26)	− 0.04 (− 0.05 to − 0.02)	< 0.001	65,664,937.65 (46,789,694.21–89,000,419.04)	123,892.14 (87,627.42–168,952.64)	0.02 (0.01 to 0.02)	< 0.001
Eastern Sub-Saharan Africa	7,547,053.35 (5,209,540.39–10,880,262.43)	7872.97 (5422.79–11,380.32)	− 1.33 (− 2.02 to − 0.64)	< 0.001	99,892.9 (68,787.38–145,154.94)	107.51 (73.63–157.16)	− 1.56 (− 2.32 to − 0.79)	< 0.001	67,054,438.81 (45,731,840.36–91,522,394.72)	67,333.97 (45,759.6–92,204.74)	− 0.06 (− 0.07 to − 0.04)	< 0.001	139,424,998.71 (104,858,490.86–179,216,322.48)	141,609.02 (106,998.48–180,851.36)	-0.01 (-0.03 to 0.01)	0.26
High-income Asia Pacific	416,123.63 (254,672.35–663,628.65)	1010.29 (603.56–1636.58)	− 0.34 (− 0.4 to − 0.28)	< 0.001	1688.08 (1552.03–1862.22)	3.25 (2.98–3.6)	− 0.81 (− 1.32 to − 0.29)	0.002	10,359,889.39 (6,560,918.56–15,264,097.17)	27,215.12 (17,290.59–39,736.33)	− 0.23 (− 0.3 to − 0.16)	< 0.001	35,460,204.17 (24,931,674.2–49,010,507.82)	88,201.09 (61,713.84–122,395.13)	-0.2 (-0.24 to -0.16)	< 0.001
High-income North America	938,892.94 (593,809.64–1,452,503.99)	1069.04 (670.65–1664.49)	− 0.74 (− 1 to − 0.48)	< 0.001	3836.62 (3674.67–4011.36)	4.1 (3.93–4.29)	− 2.22 (− 2.49 to − 1.95)	< 0.001	30,279,372.57 (19,457,880.28–44,457,371.63)	35,181.54 (22,623.17–51,496.06)	− 0.1 (− 0.19 to − 0.02)	0.02	83,905,805.23 (59,927,064.43–113,797,067.85)	96,557.74 (68,768.9–131,353.39)	0.08 (0.02 to 0.15)	0.013
North Africa and Middle East	3,365,101.26 (2,047,002.96–5,229,679.4)	2083.06 (1266.26–3240.13)	0.04 (0.02 to 0.07)	< 0.001	7932.35 (5423.48–12,913.07)	4.93 (3.37–8.03)	0.83 (0.71 to 0.96)	< 0.001	105,837,364.47 (74,857,008.29–139,161,875.05)	65,641.97 (46,370.96–86,423.63)	− 0.06 (− 0.08 to − 0.03)	< 0.001	204,515,531.42 (148,997,935.22–270,705,412.16)	126,885.27 (92,394.35–168,067.83)	0 (-0.01 to 0.01)	0.846
Oceania	73,106.02 (41,769.87–122,420.6)	2197.17 (1259.71–3674.31)	0.65 (0.24 to 1.06)	0.002	659.91 (376.66–1137.91)	20.48 (11.73–35.27)	1.21 (0.53 to 1.89)	< 0.001	2,235,713.31 (1,529,104.23–3,093,203.5)	64,622.84 (44,098.85–89,620.59)	− 0.08 (− 0.17 to 0.01)	0.079	3,894,477.02 (2,884,041.31–5,075,172.89)	113,973.85 (84,472.74–148,378.98)	0.01 (-0.01 to 0.03)	0.361
South Asia	7,791,463.05 (4,769,507.33–12,348,549.26)	1608.22 (988.26–2541.52)	− 0.19 (− 0.37 to − 0.02)	0.033	46,168.57 (33,492.04–66245.16)	9.8 (7.14–13.99)	− 0.31 (− 0.67 to 0.05)	0.092	188,772,840.52 (125,339,076.03–264177436.77)	38,390.95 (25,456.48–53,804.12)	− 0.05 (− 0.1 to − 0.01)	0.017	511,280,826.58 (356,655,734.21–703,268,393.04)	104,579.16 (73,064.77–143,647.93)	0.06 (0.05 to 0.08)	< 0.001
Southeast Asia	2,639,739.2 (1,694,076.59–4,024,105.32)	1407.64 (901.26–2150.53)	− 0.07 (− 0.2 to 0.06)	0.266	19,064.23 (14,862.55–24,049.23)	9.94 (7.75–12.54)	− 0.12 (− 0.35 to 0.11)	0.289	64,093,479.46 (42,388,515.03–90247603.54)	34,682.01 (22,940.29–48,781.74)	− 0.12 (− 0.15 to − 0.09)	< 0.001	182,982,291.37 (129,666,488.17–248,802,215.24)	98,490.58 (69,706.07–134077.53)	0.05 (0.02 to 0.07)	< 0.001
Southern Latin America	308,923.82 (203,294.12–459,491.19)	1703.18 (1117.36–2540)	− 0.16 (− 0.32 to 0)	0.046	1920.46 (1674.63–2207.27)	10.34 (9.02–11.88)	− 0.5 (− 0.86 to − 0.14)	0.007	7,202,150.63 (4,711,728.31–10,192,071.89)	40,452.97 (26,477.93–57,228.65)	− 0.01 (− 0.02 to 0)	0.012	20,254,016.59 (14,521,458.83–27,382,483.06)	112,965.12 (80,853.5–152,992.06)	-0.04 (-0.06 to -0.03)	< 0.001
Southern Sub-Saharan Africa	3,999,401.08 (3,334,706.18–4,911,511.09)	18,363.82 (15,293.01–22580.62)	2.67 (1.73 to 3.62)	< 0.001	56,189.27 (47,335.79–68,941.77)	259.4 (218.15–318.88)	3.04 (1.96 to 4.14)	< 0.001	18,778,266.98 (12,956,669.58–25,409,180.46)	85,937.98 (59,243.64–116,538.74)	− 0.18 (− 0.22 to − 0.14)	< 0.001	39,391,163.04 (30,939,387.82–49,210,251.24)	181,440.51 (142,664.6–226,325.06)	0.33 (0.31 to 0.36)	< 0.001
Tropical Latin America	1,062,580.24 (761,867.07–1500363.65)	1666.43 (1188.13–2364.65)	− 0.04 (− 0.21 to 0.13)	0.612	8300.24 (7904.55–8718.72)	12.6 (12.01–13.24)	− 0.03 (− 0.22 to 0.17)	0.801	31,546,155.02 (21,346,663.31–44,116,537.11)	50,772.06 (34,354.4–70,895.65)	− 0.09 (− 0.14 to − 0.04)	< 0.001	76,163,488.91 (56,917,825.91–99,071,480.58)	121,056.88 (90,243.89–157,803.43)	-0.07 (-0.09 to -0.05)	< 0.001
Western Europe	1,633,913.9 (1,001,677.1–2,543,520.37)	1639.73 (996.35–2571.32)	− 0.48 (− 0.53 to − 0.43)	< 0.001	3249.33 (3054.94–3459.07)	2.83 (2.66–3.01)	− 2.52 (− 2.92 to − 2.12)	< 0.001	44,111,765.43 (29,621,725.69–60,564,926.17)	45,686.89 (30,750.19–62,709.29)	− 0.23 (− 0.25 to − 0.2)	< 0.001	105,258,962.37 (73,420,023.76–144,067,139.47)	107,025.83 (74,492.49–147,041.07)	-0.13 (-0.15 to -0.1)	< 0.001
Western Sub-Saharan Africa	5,559,641.24 (3,687,543.7–8,125,169.2)	5348.22 (3588.05–7733.92)	0.53 (0.13 to 0.93)	0.01	66,801.18 (45,519.39–95,420.3)	66.67 (45.94–94.22)	0.91 (0.33 to 1.49)	0.002	74,394,959.6 (50,740,737.18–101,638,066.12)	67,788.77 (46,209.95–92,661.58)	0.01 (− 0.01 to 0.03)	0.252	145,562,758.62 (107,092,617.56–191,157,366.3)	134,113.49 (99,258.63–174,833.53)	0 (-0.01 to 0.01)	0.998

*DALYs* Disability-adjusted life-years, *ASDR* Age-standardized DALYs rate, *ASMR* Age-standardized mortality rate, *ASIR* Age-standardized incidence rate, *ASPR* Age-standardized prevalence rate, *CI* Confidence interval, *AAPC* Average annual percentage change

The details of different types of gynecological disorders are summarized in Table 2. In 2021, HIV/AIDS was the most common cause of DALYs and death worldwide (16.38 million and 0.27 million, respectively). Other three common causes of DALYs included PMS (7.43 million), cervical cancer (4.18 million), and endometriosis (1.94 million), except for other gynecological diseases. In terms of incidence, the leading disorders were sexually transmitted infections excluding HIV (286.51 million), PMS (232.6 million), and uterine fibroids (9.83 million). PMS (889.97 million) represented the most significant proportion of CGDs prevalence, accounting for approximately 41%. Despite most diseases exhibited a decline in ASDR (AAPC < 0, P < 0.05), HIV/AIDS (AAPC = 1.6%), PCOS (0.79%), female infertility (0.68%), PMS (0.04%) contributed the overall increase for CGDs. The most significant increases for ASIR were observed in PCOS (0.7%) and uterine cancer (0.54%).

**Table 2:** Global burden of different types of gynecological diseases among women of childbearing age in 2021 and their average annual percentage changes from 1990 to 2021

Disorders	DALYs (95% CI)				Death (95% CI)				Incidence (95% CI)				Prevalence (95% CI)			
	Count	ASDR (per 100,000 population)	AAPC for ASDR (%)	*P-value*	Count	ASMR (per 100,000 population)	AAPC for ASMR (%)	*P-value*	Count	ASIR (per 100,000 population)	AAPC for ASIR (%)	*P-value*	Count	ASPR (per 100,000 population)	AAPC for ASPR (%)	*P-value*
HIV/AIDS	16,378,796.14 (13,002,587.28–20,997,995.28)	829.75 (658.24–1063.9)	1.6 (1.14 to 2.07)	< 0.001	265,696.44 (205,247.78–348,436.39)	13.39 (10.34–17.56)	1.58 (1.09 to 2.06)	< 0.001	670,804.13 (579,764.81–782,486.58)	34.73 (30.03–40.54)	− 1.93 (− 2.22 to − 1.65)	< 0.001	16,497,874.15 (15,584,846.64–17,544,995)	830.87 (784.57–884.29)	3.85 (3.48 to 4.22)	< 0.001
Sexually transmitted infections excluding HIV	700,302.55 (416,101.03–1170891.54)	35.45 (21.1–59.17)	− 0.15 (− 0.18 to − 0.11)	< 0.001	2215.02 (1345.93–3147.82)	0.11 (0.07–0.16)	− 1.28 (− 1.48 to − 1.08)	< 0.001	286,514,651.39 (203,966,590.72–390,280,447.3)	14,579.13 (10,398.78–19,805.4)	0.13 (0.05 to 0.21)	0.002	544,454,239.16 (462,796,611.55–636,018,560.96)	27,477.76 (23,342.43–32,118.6)	0.12 (0.08 to 0.16)	< 0.001
Premenstrual syndrome	7,427,207.92 (4,148,170.82–12,131,572.89)	379.98 (212.13–621.53)	0.04 (0.03 to 0.05)	< 0.001	NA	NA	NA	NA	232,596,759.97 (143,119,886.08–330272130.4)	11,993.65 (7373.61–17,033.12)	0.04 (0.03 to 0.06)	< 0.001	889,970,473.02 (618,097,378.23–1,185,904,888.28)	45,504.28 (31,591.54–60,679.05)	0.04 (0.03 to 0.05)	< 0.001
Endometriosis	1,939,179.55 (1,043,871.12–3,250,764.22)	98.69 (53.12–165.44)	− 0.98 (− 1.03 to − 0.94)	< 0.001	46.72 (16.87–106.87)	0 (0–0.01)	0.8 (0.31 to 1.29)	0.001	3,439,457.24 (1,795,773.54–5,603,240.06)	178.52 (93.66–290.2)	− 0.97 (− 1 to − 0.93)	< 0.001	21,045,890.51 (12,874,335.18–31,892,235.3)	1070.7 (654.43–1623.79)	− 0.98 (− 1.03 to − 0.94)	< 0.001
Female infertility	601,133.5 (124,293.13–1,716,290.98)	30.55 (6.32–87.18)	0.68 (0.58 to 0.78)	< 0.001	NA	NA	NA	NA	NA	NA	NA	NA	110,089,459.27 (31,051,019.4–254,716,760.03)	5586.19 (1580.25–12,917.2)	0.66 (0.57 to 0.76)	< 0.001
Polycystic ovarian syndrome	576,045.19 (255,576.76–1,200,184.51)	29.51 (13.09–61.49)	0.79 (0.75 to 0.84)	< 0.001	NA	NA	NA	NA	1,175,074.43 (711,336.35–1,887,245.89)	64.44 (39.07–103.4)	0.7 (0.67 to 0.72)	< 0.001	65,767,552.9 (46,839,857.45–91,498,217.36)	3364.53 (2395.08–4681.81)	0.81 (0.77 to 0.85)	< 0.001
Uterine fibroids	99,024.6 (66,843.42–141,126.74)	4.92 (3.32–7.02)	0.01 (− 0.15 to 0.16)	0.948	996.98 (586.28–1318.93)	0.05 (0.03–0.07)	− 0.28 (− 0.43 to − 0.13)	< 0.001	9,830,184.7 (4,977,545.72–16,246,619.07)	494.22 (249.78–817.56)	0.25 (0.21 to 0.28)	< 0.001	85,180,728.48 (56,514,987.95–122,225,847.11)	4221.75 (2797.57–6064.56)	0.17 (0.14 to 0.21)	< 0.001
Genital prolapse	101,961.39 (46,950.67–209,482.13)	5.01 (2.3–10.3)	− 0.77 (− 0.81 to − 0.73)	< 0.001	20.74 (12.45–29.5)	0 (0–0)	− 1.13 (− 1.34 to − 0.93)	< 0.001	5,629,892.91 (3,416,878.3–8,480,665.02)	277.83 (168.76–418.44)	− 0.64 (− 0.67 to − 0.62)	< 0.001	32,019,655.76 (21,885,504.05–44438602.12)	1571.79 (1073.15–2184.28)	− 0.78 (− 0.81 to − 0.74)	< 0.001
Cervical cancer	4,184,314.17 (3,751,959.2–4,659,907.95)	206.68 (185.28–230.25)	− 1.2 (− 1.33 to − 1.08)	< 0.001	81,639.89 (73,425.84–90,920.78)	4.02 (3.61–4.47)	− 1.26 (− 1.38 to − 1.13)	< 0.001	307,427.99 (278,346.99–338,666.85)	15.21 (13.77–16.76)	− 0.4 (− 0.49 to − 0.31)	< 0.001	2,046,773 (1,858,237.66–2,254,921.98)	101.49 (92.14–111.81)	− 0.01 (− 0.11 to 0.09)	0.855
Uterine cancer	373,681.68 (308,480.56–426,329)	18.29 (15.09–20.88)	− 1.02 (− 1.23 to − 0.82)	< 0.001	7163.36 (5927.45–8139.5)	0.35 (0.29–0.4)	− 1.12 (− 1.32 to − 0.92)	< 0.001	58,860.14 (50,122.62–66,326.97)	2.87 (2.44–3.23)	0.54 (0.31 to 0.78)	< 0.001	483,867.67 (412,704.51–544,244.39)	23.59 (20.11–26.54)	0.64 (0.35 to 0.92)	< 0.001
Ovarian cancer	1,294,995.57 (1,125,255.2–1,446,127.87)	64.04 (55.59–71.56)	− 0.2 (− 0.32 to − 0.07)	0.002	25,257.83 (22,047.49–28,131.91)	1.24 (1.08–1.38)	− 0.26 (− 0.39 to − 0.12)	< 0.001	85,748.81 (74,200.59–95,797.26)	4.27 (3.69–4.78)	0.23 (0.13 to 0.33)	< 0.001	522,318.7 (452,336.73–584,164.21)	26.14 (22.61–29.26)	0.42 (0.33 to 0.51)	< 0.001
Benign and in situ cervical and uterine neoplasms	NA	NA	NA	NA	NA	NA	NA	NA	491,979.2 (315,141.32–729,851.7)	24.81 (15.89–36.82)	0.34 (0.11 to 0.57)	0.004	1,373,743.89 (926,463.55–1,919,949.3)	68.54 (46.19–95.78)	0.15 (− 0.12 to 0.42)	0.272
Other gynecological diseases	11,541,889.1 (7,025,556.83–17,560,389.09)	577.82 (351.42–879.75)	− 0.12 (− 0.15 to − 0.1)	< 0.001	2676.82 (1690.8–3388.68)	0.14 (0.09–0.17)	0.28 (0.14 to 0.41)	< 0.001	358,267,612.4 (252,575,236.83–477,616,931.37)	18,020.33 (12,705.37–24,031.07)	− 0.11 (− 0.13 to − 0.09)	< 0.001	400,683,775.69 (294,902,854.99–524,342,218.88)	20,089.73 (14,773.4–26,322.7)	− 0.09 (− 0.11 to − 0.07)	< 0.001

*DALYs* Disability-adjusted life-years, *ASDR* Age-standardized DALYs rate, *ASMR* Age-standardized mortality rate, *ASIR* Age-standardized incidence rate, *ASPR* Age-standardized prevalence rate, *CI* Confidence interval, *AAPC* Average annual percentage change

According to the most recent data from GLOBOCAN 2022, the global burden of female cancers continued to present public health challenges in 2022. Cervical cancer emerged as a leading contributor, with 255,218 new cases and 93,850 deaths reported worldwide, resulting in ASIR of 12.5 per 100,000 and ASMR of 4.6 per 100,000. Uterine cancer also played a significant role in the female cancers burden, which had 60,766 new cases and 6,364 deaths globally in 2022, with ASIR of 3.0 and ASMR of 0.32 per 100,000. Ovarian cancer saw 89,948 new cases and 32,130 deaths, with ASIR of 4.5 and ASMR of 1.6 per 100,000.

### Regional and national burden of gynecological diseases in WCBA

Regional CGDs burden varied significantly. More specially, Australasia showed the lowest ASMR (per 100,000 population) at 2.37, while Southern Sub-Saharan Africa peaked at 259.4—approximately 109-fold higher than the minimum (Table 1). High-income Asia Pacific showed the lowest ASDR (1010.29/100,000), compared to Southern Sub-Saharan Africa's highest (18,363.82/100,000)—about 18 times higher. Southern Sub-Saharan Africa also led in ASIR and ASPR, with the lowest in High-income Asia Pacific and Central Europe, respectively. From 1990 to 2021, Southern Sub-Saharan Africa showed the largest rise in ASDR (AAPC = 2.67%[1.73% to 3.62%]), ASMR (3.04%[1.96% to 4.14%]), and ASPR (0.33%[0.31% to 0.36%]) (Table 1).

**Fig. 2:**
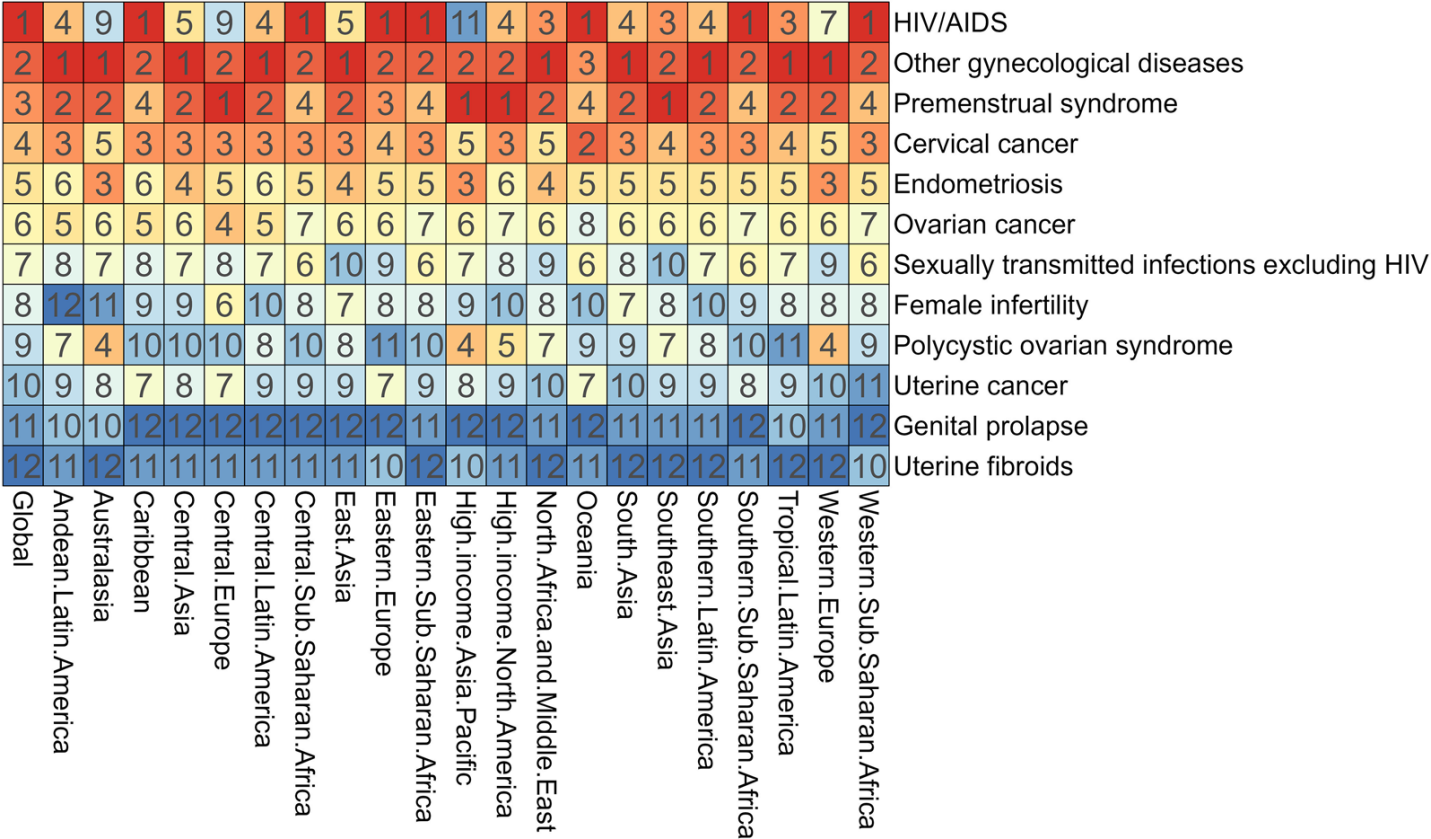
Ranking of age-standardized DALYs rates for different types of gynecological disorders among women of childbearing age by GBD region in 2021

Regional ranks of different gynecological diseases burdens among WCBA in 2021 are described in Fig. 2 and Supplementary Figs. 1–3. HIV/AIDS, other gynecological diseases, PMS, cervical cancer, and endometriosis were top five conditions by ASDR in 13 of 21 GBD regions. Regional ranking of ASDR across disorders were mostly consistent, with exceptions for HIV/AIDS (regional ranking range: first to 11th) and PCOS (4th to 11th). In particular, the highest ASDR (per 100,000 population) were reported in Southern Sub-Saharan Africa for HIV/AIDS (15,941.34), cervical cancer (725.28) and uterine fibroids (14.56), while South Asia had the highest for PMS (417.3), Oceania for endometriosis (175.16), Eastern Europe for ovarian cancer (104.56), High-income Asia Pacific for PCOS (88.17), Caribbean for uterine cancer (62.39), and East Asia for female infertility (43.54) (Tables S2-S14).

**Fig. 3:**
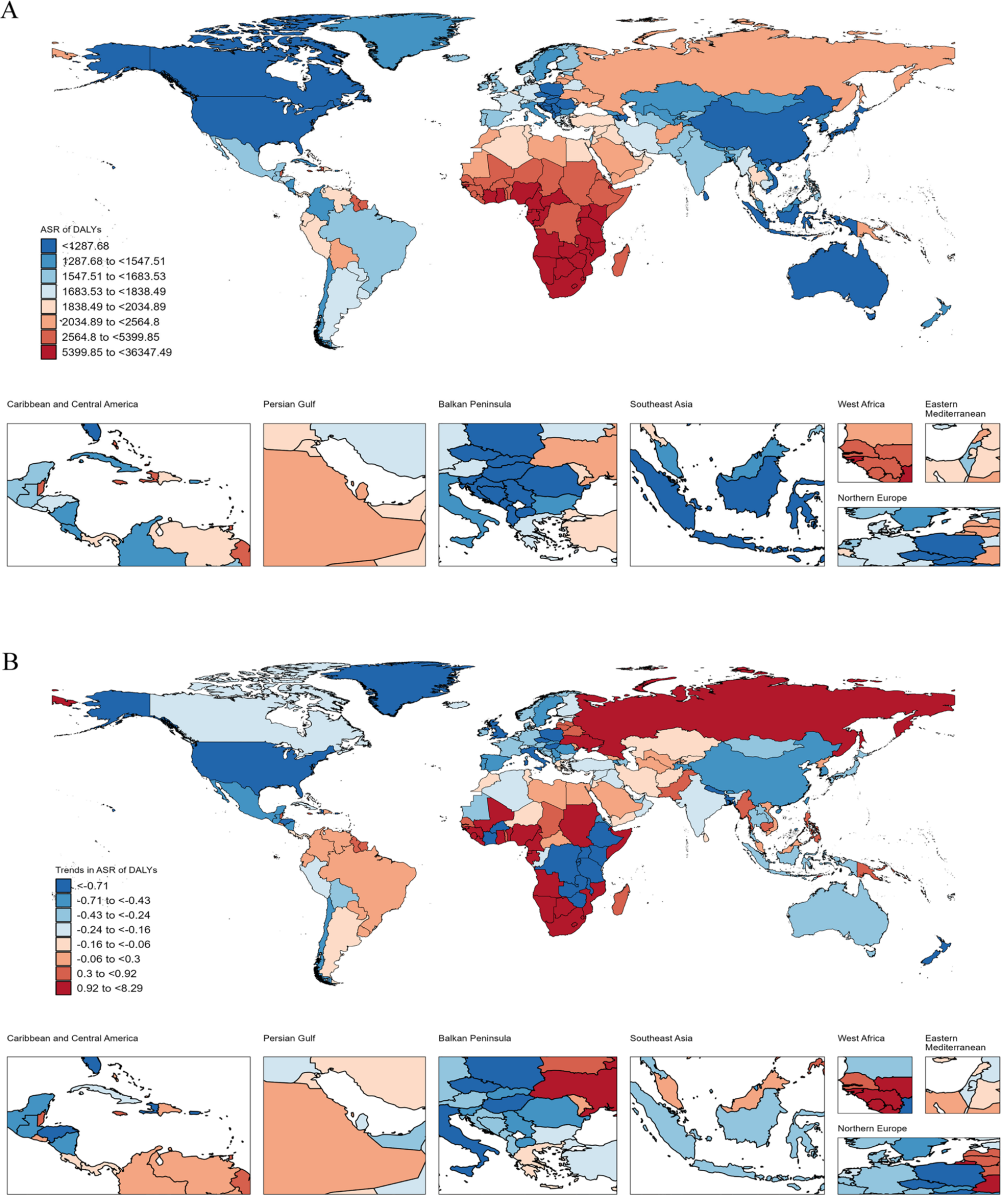
National age-standardized DALYs rates of combined gynecological disorders (CGDs) among women of childbearing age in 2021 and their average annual percentage change from 1990 to 2021. **A** Age-standardized DALYs rates; **B** AAPC for age-standardized DALYs rates. *DALYs* Disability-Adjusted Life Years, *AAPC* Average annual percentage change. Map lines delineate study areas and do not necessarily depict accepted national boundaries, which however was not the key point for this study

At the national level, in 2021, Lesotho, Eswatini, Mozambique, Botswana, and Equatorial Guinea had the highest ASDR and ASMR in CGDs in WCBA, while South Africa, Lesotho, Eswatini, and Botswana reported the highest ASIR and ASPR (Tables S1 and Fig. 3). From 1990 to 2021, 51 countries experienced upward in ASDR, 58 countries had rising in ASMR, and 102 countries showed increasing in ASPR, with Eswatini showing the largest increases in ASDR (AAPC, 8.29%[7.03% to 9.57%]), ASMR (11.27%[9.67% to 12.9%]), and ASPR (0.71%[0.67% to 75%]). Additionally, 24 countries experienced rising in ASIR, with Taiwan (Province of China) having the largest rise (0.12%[0.1% to 0.14%]) (Tables S1 and Figures S4-6).

### The association between gynecological diseases burden and SDI

**Fig. 4:**
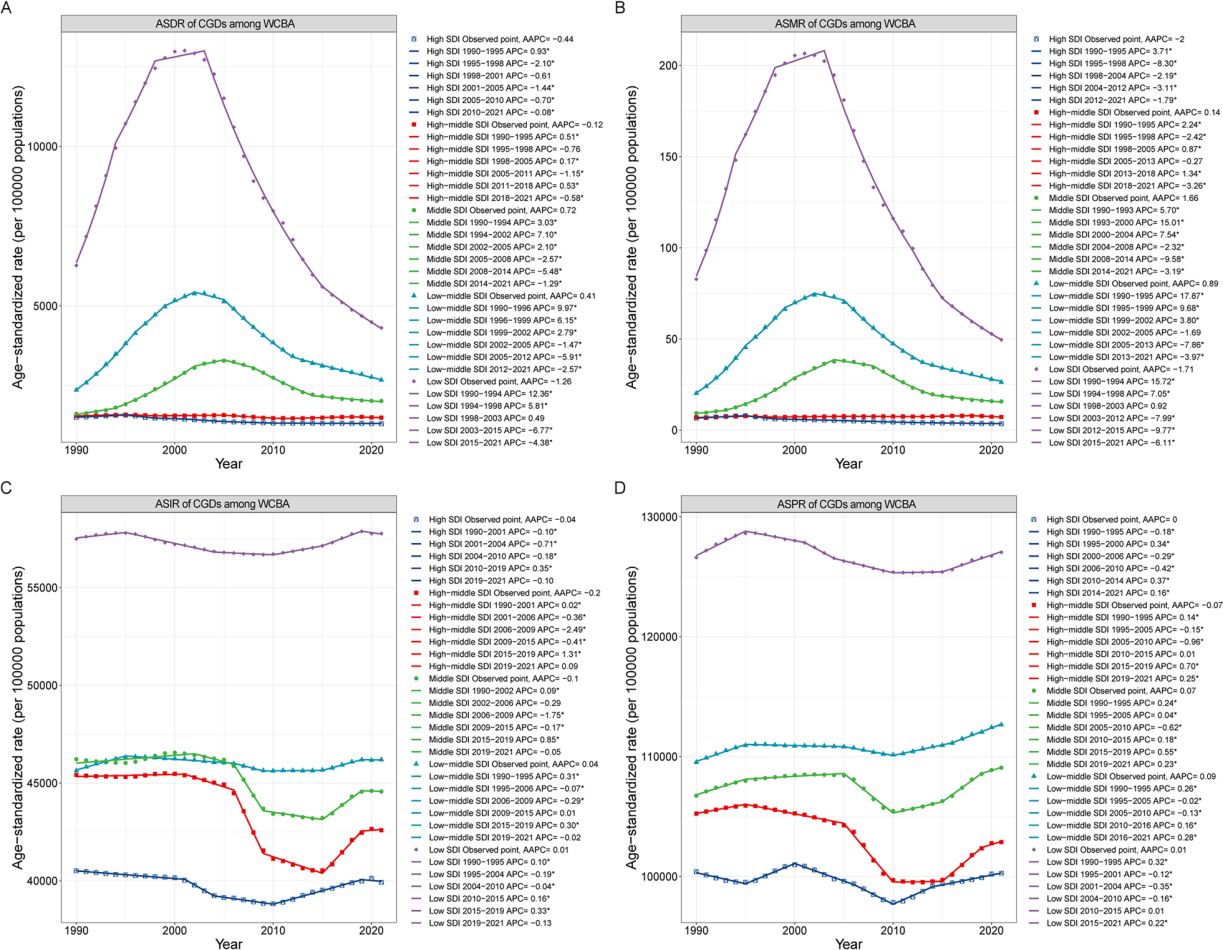
Trends of combined gynecological disorders (CGDs) burden in women of childbearing age among different SDI quintiles from 1990 to 2021. **A** Age-standardized DALYs rate; **B** Age-standardized mortality rate; **C** Age-standardized incidence rate; **D** Age-standardized prevalence rate. * Indicates that the Annual Percent Change (APC) is significantly different from zero at the alpha = 0.05 level. *SDI* Socio-demographic index, *DALYs* Disability-adjusted life-years

The SDI serves as a proxy for the quality and accessibility of healthcare across various nations. As shown in Table 1 and Fig. 4, the burden of CGDs varied markedly by SDI. In 2021, countries with low SDI recorded the highest ASDR (4309.35), ASMR (49.54), ASIR (57,770.87), and ASPR (127,041.23), while nations with high SDI displayed the lowest ASDR (1304.84), ASMR (3.54), ASIR (39,916.14), and ASPR (100,274.97). Since 1990 to 2021, low-middle SDI and middle SDI countries showed rising in ASDR and ASMR, with middle SDI countries having the largest increase (ASDR: AAPC = 0.72%[0.46% to 0.98%]; ASMR: AAPC = 1.66%[1.24% to 2.08%]).

**Fig. 5:**
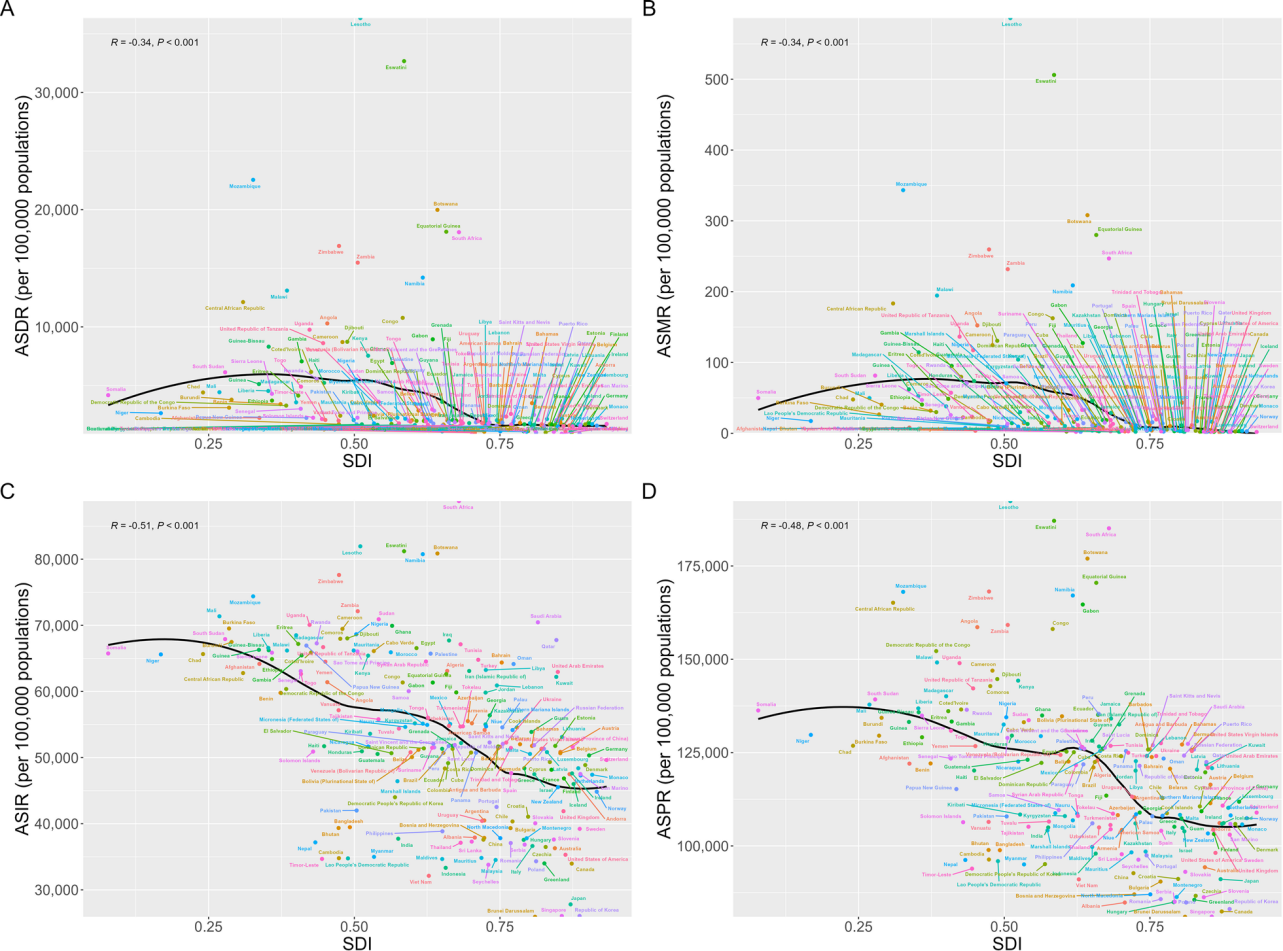
The association between SDI and combined gynecological disorders (CGDs) burden among women of childbearing age for 204 countries and territories in 2021. **A** Age-standardized DALYs rate; **B** Age-standardized mortality rate; **C** Age-standardized incidence rate; (D) Age-standardized prevalence rate. Abbreviations: SDI, socio-demographic index, DALYs, disability-adjusted life-years

To explore the relationship between SDI and CGDs burdens, we performed Pearson correlation analysis (Fig. 5). Expected values based on the SDI and ASR in all locations are shown as the black line. Each point shows the observed ASR for each country in 2021. Countries above the solid black line represent a higher than expected burden and countries below the line show a lower than expected burden. Negative correlations existed between CGDs burden and SDI: ASDR (*R* = − 0.33, *P* < 0.001), ASMR (*R* = − 0.34, *P* < 0.001), ASIR (*R* = − 0.50, *P* < 0.001), and ASPR (*R* = − 0.47, *P* < 0.001), meaning that the higher the SDI in a country, the lower CGDs burden. Notably, within the SDI value range of 0.5 to 0.75, the decline of burden was more pronounced with increasing SDI values.

According to Table S15, in the analysis of the ASDR, ASMR, ASIR, and ASPR for sexually transmitted infections, as well as endometriosis, genital prolapse, and other gynecological diseases, a negative correlation was observed with SDI across 204 countries, while PCOS showed moderate positive correlations. For gynecological cancers, SDI negatively correlated with these indicators in cervical cancer but positively in ovarian cancer.

### Age subgroup differences in gynecological diseases burden

**Fig. 6:**
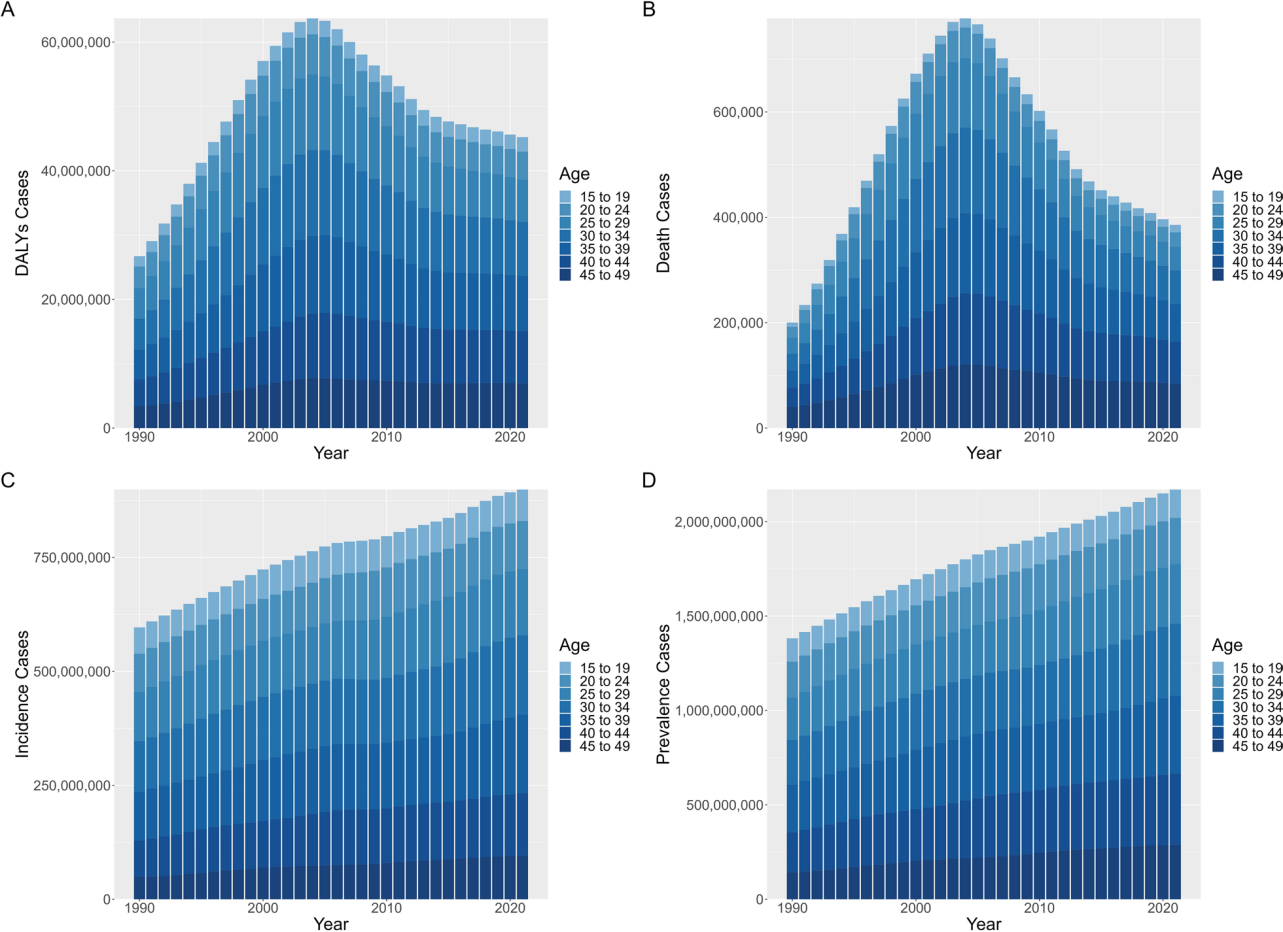
Burdens of combined gynecological disorders (CGDs) among women of childbearing age from 1990 to 2021 for different age groups. (A)DALYs; **B** Death; **C** Incidence; **D** Prevalence. *DALYs* Disability-adjusted life-years

Among different age groups, women aged 35–39 years had the highest CGDs DALYs (8.62 million) and prevalence (413.17 million) in 2021, while women aged 45–49 years had the most death (83.83 thousands) and those aged 30–34 had the highest incidence (174.69 million) (Table 1 and Fig. 6).

Most age groups saw increases in age-specific rates of CGDs from 1990 to 2021 (Table 1). Detailly, the highest rises in women aged 15–19 years for DALYs rate (AAPC = 0.52%[0.42% to 0.61%]) and death rate (1.2%[0.89% to 1.52%]), while the most significant rise in women aged 20–24 years for incidence rate (0.13%[0.11% to 0.16%]) and prevalence rate (0.25%[0.24% to 0.27%]), indicating the CGDs burdens are increasingly affecting younger women. To explore the underlying causes, we compared the growth trends of different gynecological diseases across age groups. Some conditions showed the fastest growth in women aged 15–19 years, such as PCOS with an AAPC of 0.79% for ASIR and other gynecological diseases exhibited an AAPC of 0.82% for ASDR, while infertility in women aged 20–24 years had an AAPC of 1.04% for ASPR.

**Fig. 7:**
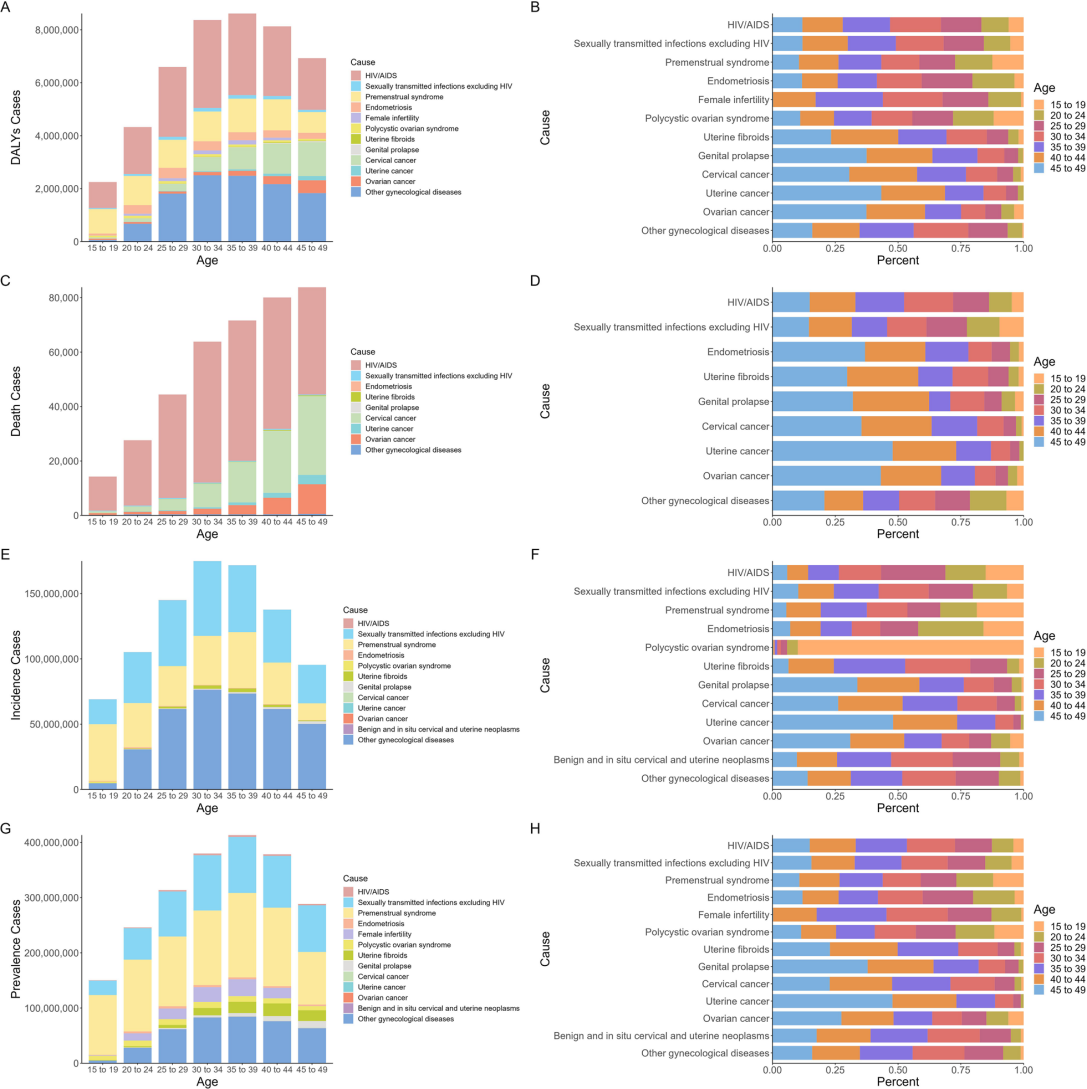
Burdens of different types of gynecological diseases for different age groups and their proportions in 2021. **A** DALYs; **B** Proportions in DALYs. **C** Death; **D** Proportions in death; **E** Incidence; **F** Proportions in incidence; **G** Prevalence; **H** Proportions in prevalence. *DALYs* Disability-adjusted life-years

Influenced by factors such as lifestyle changes, healthcare access, and genetic predispositions, the DALYs and mortality rates for gynecological disorders varied across different age groups, as demonstrated in Fig. 7. Women aged in 40–49 years faced the highest from malignant tumors, genital prolapse, and uterine fibroids (proportions ranged from 50.08%-73.14%), while 30–39 years were most affected by sexually transmitted infections. The 15–19 age group represented approximately 89.9% of new PCOS cases, 20–24 age group was 25.99% of incidence of endometriosis, and 30–39 accounted for 52.2% of prevalence in infertility. Women aged 45–49 years constituted the primary demographic for both incidence and prevalence of uterine cancer (48.01%, 47.77%), Genital prolapse (33.8%, 37.9%) and Ovarian cancer (30.97% and 27.44%). This age-specific distribution underscores the varied impact of gynecological conditions and the necessity for targeted health strategies across different life stages.

### Projections of gynecological diseases burdens up to 2031

**Fig. 8:**
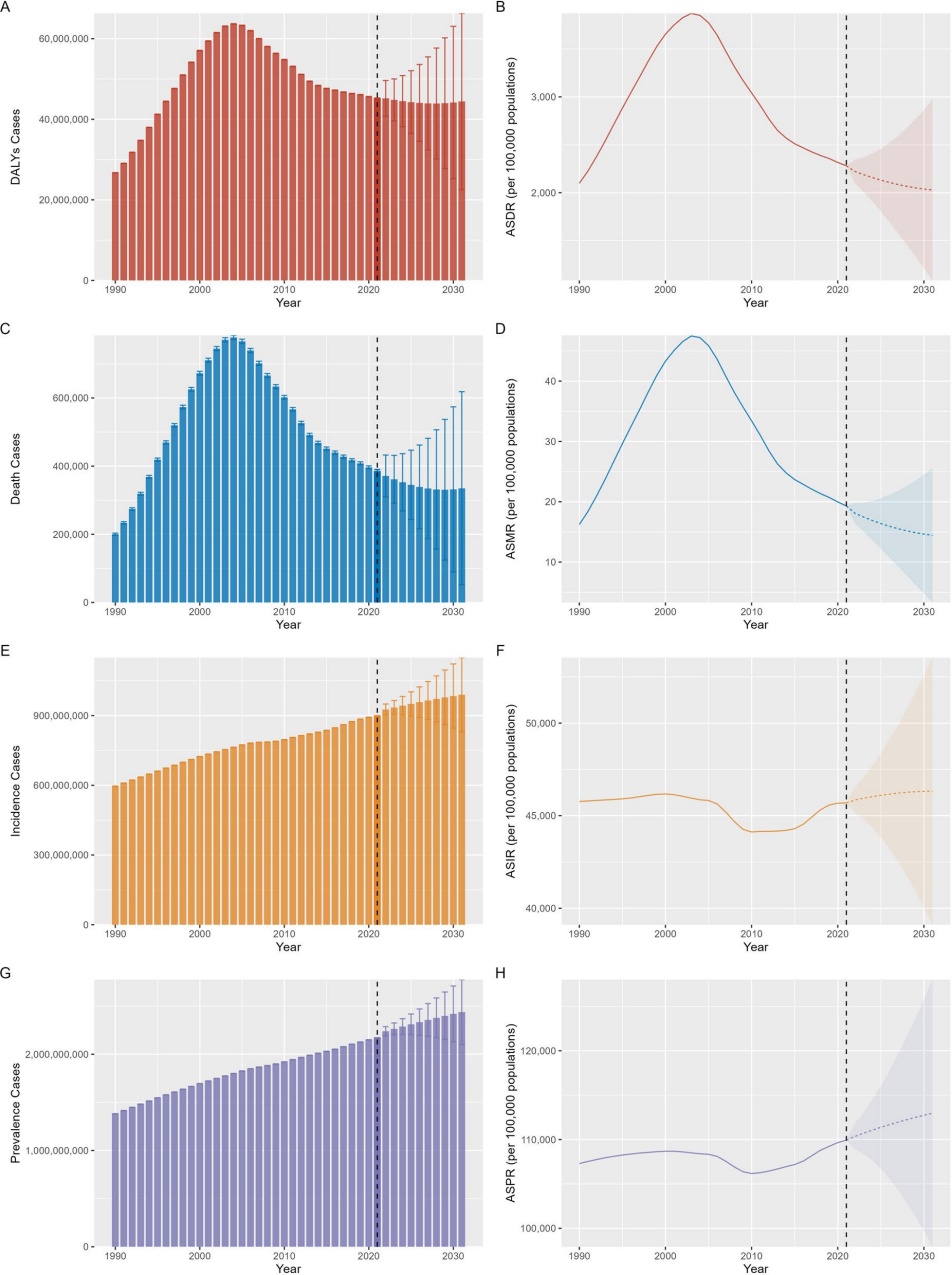
Projections of combined gynecological diseases (CGDs) burden in women of childbearing age by 2031 based on the Bayesian age-period-cohort (BAPC) models. **A** The predicted case number of DALYs; **B** The predicted age-standardized DALYs rates; **C** The predicted case number of death; **D** The predicted age-standardized mortality rates; **E** The predicted case number of incidence; **F** The predicted age-standardized incidence rates; **G** The predicted case number of prevalence; **H** The predicted age-standardized prevalence rates. *DALYs* Disability-adjusted life-years

According to BAPC model, ASDR and ASMR of CGDs were predicted to reduction in next decade with − 11.06% and − 25.23%, respectively. Conversely, the ASIR and ASPR were anticipated to rise by 1.39% and 2.75%, which likely due to improvements in screening and treatment (Table S16 and Fig. 8). The analysis results based on the Nordpred model also proved the same conclusion: the ASDR and ASMR of CGDs were predicted to decreased by − 10.52% and − 22.94%, whereas the ASIR and ASPR were anticipated to increased by 3.38% and 2.73% (Table S17). The reason for the larger increase observed in the BAPC model may be that the Nordpred model analysis uses the average of the five-year data. This averaging effect tends to obscure the magnitude of increase, rendering it less apparent in comparison to the results derived from the BAPC model analysis.

Regarding projections for various gynecological diseases, the most significant increases were observed for PCOS (ASIR: 15.03%; ASPR: 10.31%; ASDR: 10.03%) and female infertility (ASPR: 11.54%; ASDR: 10.83%) (Table S16 and Figure S7-8). In contrast, HIV/AIDS was anticipated to see the most notable declines in ASIR (− 51.5%), ASMR (− 26.66%), and ASDR (− 22.82%) by 2031. The same predicted trends was obtained using the Nordpred model (Table S17).

## Discussion

To the best of our knowledge, this study provides the latest and most comprehensive epidemiological estimates of gynecological disorders burdens in women of childbearing age at the global, regional, and national levels during 1990 to 2021, integrating a multitude of factors including SDI and age. In 2021, CGDs accounted for 12.34% of all cause death cases in WCBA worldwide, underscoring the substantial impact of gynecological diseases on public health. Over the last 32 years, there were significant increases in the ASDR, ASMR, and ASPR of CGDs, while ASIR remained stable. Regarding age differences, women aged 15–24 years exhibited the most significant increase in CGDs burdens, highlighting its growing impact on younger women. CGDs burdens were negatively correlated with and SDI. Encouragingly, the BAPC model forecasts that the global ASDR and ASMR of CGDs may decrease in the coming decade, despite the predictions of a rise in ASIR and ASPR.

Between 1990 and 2021, our study indicates a global increase in ASDR, ASMR, and ASPR for CGDs, probably attributable to population growth, aging, effective therapies, and improved life expectancy. This findings diverges from previous study, likely due to differences in the time period, age groups and categories of gynecological diseases investigated [11]. Most gynecological disorders were inversely correlated with SDI, whereas PCOS and ovarian cancer demonstrated a positive correlation, consistent with previous studies [9], due to higher obesity rates more common in developed countries with westernized diets [28]. Additionally, factors like less breastfeeding, infertility, and hormone treatment, are linked to ovarian cancer, while oral contraceptive use offer protection [29].

Our results showed that the burden of CGDs varied geographically, with low SDI countries still having the highest burdens. Furthermore, only low-middle and middle SDI nations exhibited an increase in ASDR and ASMR, highlighting the need for more focus on their health challenges. The heavy burdens in underdeveloped areas, attributed to multifaceted factors, including poverty, restricted access to healthcare, and inadequate early diagnosis and treatment [30, 31].

The age distribution of different gynecological diseases varies greatly. On one hand, women aged 15–19 years accounted for a considerable proportion and increased significantly in the incidence of PCOS, similar to previous study [11]. PCOS is a reproductive and metabolic disorder that affects individuals across their lifespan, leading to issues such as anxiety, eating disorders, psychosexual dysfunction, and increased risks of pregnancy complications and endometrial cancer [32]. The rise in obesity among girls in recent decades has increased PCOS incidence in adolescents, mainly due to dietary and lifestyle changes like lack of exercise and consumption of processed foods and sugary drinks [33]. Therefore, the inclusion of dietary and exercise interventions in schools and communities is essential for promoting healthier lifestyles, particularly in areas with high BMI among adolescent. On the other hand, women aged 45–49 years had the highest proportion of genital prolapse, vaginal delivery and estrogen decline were the most important factor, more apparent as women age [34].

The projections indicated that the global ASDR and ASMR of CGDs may decrease over the following decade. Prevention strategies and advancements in medical treatments, coupled with lifestyle modifications, may partially contribute to the downward trends [35]. However, the improving in diagnostic and screening may lead to a rise in ASIR and ASPR, as more cases are identified.

Although the majority of diseases showed a reduction in global ASDR, HIV/AIDS, PCOS, infertility, and PMS emerged as the primary contributors to the heightened ASDR of CGDs. UNAIDS has set the goals of "End Inequalities. End AIDS" for 2021–2026 and aims to eliminate AIDS by 2030 [36]. However, HIV/AIDS burdens remain highest in Southern Sub-Saharan Africa, especially in low SDI countries where needing expanded antiretroviral therapy (ART) coverage. Additionally, the increasing gender disparity in HIV incidence, especially among women [37], underscores the impact of stigma, gender inequality, and partner violence, necessitating policies for economic and gender-transformative interventions to improve health outcomes.

Regarding female infertility burden, it has been observed increasing over the past 32 years, and projected to continue rising in the next decade. The causes of female infertility are multifaceted, with the most common being ovulatory dysfunction, diminished ovarian reserve, tubal disease, and factors related to the uterus and cervix. Gender inequality further exacerbates the plight of women with infertility, who more likely to face domestic violence, social stigma, emotional stress, and depression [38]. Although assisted reproduction technologies (ART) have existed for over the last four decades and resulting in over 8 million births since 1978, these technologies remain largely inaccessible and unaffordable in many regions, particularly in low and middle-income countries (LMICs) [39]. For instance, in Brazil, access to services related to ART and fertility preservation is neither easy nor fair, with publicly funded treatment remaining scarce and thus deepening social disparities [40]. Policy makers and healthcare providers should investigate the factors contributing to female infertility and enhance the availability and fairness of care and treatment for this condition, particularly in low- and middle-income countries.

PMS emerges as a predominant health concern among WCBA, accounting for approximately 41% of CGDs, indicating a slight upward trend globally. Characterized by late luteal phase disorder, PMS manifests as a combination of physical, psychological, and social symptoms before menstruation, affecting women's quality of life and potentially causing sleep disturbances, postpartum depression, and hypertension [41]. Factors like adiposity, stress, smoking, high sugar intake, and heredity possibly increase risk of PMS [42]. These risk factors not included in the GBD 2021 study, and further research is required to assess the association. Consistent with previous findings, we found that PMS burden highest in women aged 35–39 years, likely due to the increased social and marital responsibilities [10]. Meanwhile, the incidence rate of PMS was also high in adolescents, as their immaturity of the hypothalamic-pituitary–gonadal (HPG) axis and sensitivity to hormonal changes [43].

Our study reveals an increasing burden of CGDs among WCBA worldwide, with significant disparities by SDI and age. Therefore, it is essential to explore contributed causes and risk factors, particularly among younger women. Additionally, future research should investigate the role of lifestyle, environmental factors, and genetic predispositions in the development of these conditions.

For cervical cancer, we found that its global burden in WCBA was decline over the past 32 years. Characterized by slow progression and early detectable precancerous lesions, cervical cancer is the most preventable cancer, primarily prevention through HPV vaccination and screening initiatives [44]. In 2020, WHO launched the global Cervical Cancer Elimination Initiative to accelerate the elimination of cervical cancer, with the objective of reducing incidence below 4 cases per 100,000 women-years, thereby addressing the international disparity related to the disease [45]. Nevertheless, the Southern Sub-Saharan Africa region endured the greatest cervical cancer burden due to inadequate diagnostic and therapeutic services [31]. High SDI countries exhibited the most pronounced decline in ASDR, attributed to recent increases in the uptake of HPV vaccination and cervical cancer screening primarily [46].

Compared with the previous GBD study of individual gynecological diseases burden, we provide a more specific, comprehensive and updated analysis of common gynecological diseases, facilitating a more holistic evaluation of reproductive health in WCBA. Additionally, we utilized an array of statistical models, encompassing traditional models like the joinpoint regression model and innovative models such as the BAPC model. However, this study has several limitations. Firstly, gynecological disorders comprise a broad spectrum of conditions characterized by diverse diagnostic criteria, lacking uniformity. The diagnosis of certain diseases relies on subjective judgment of health care providers, which may lead to diagnostic biases. The included disorders of our analysis may not be exhaustive, which may lead to underestimation of CGDs burden. Secondly, our study relies solely on the GBD 2021, making result accuracy dependent on its data quality and quantity. The GBD study offers comprehensive estimates of disease burden but has biases, especially underreporting in LICs. The hierarchical Bayesian model integrates global-regional-country or regional disease burden, using data from neighboring areas to fill the data gap. Thus, our secondary analysis used complete data, though it has inherent defects and may not reflect the true values. The results in LMICs and LICs should be interpreted with caution due to challenges in original data collection and inadequate quality assurance. Finally, the information of attributable risk factors associated with gynecological diseases provided by GBD 2021 is limited; however, we conducted detailed analysis in discussion.

## Conclusion

In conclusion, our study provides a comprehensive evaluation of global CGDs burdens in WCBA. The global burden of CGDs has been increasing during the past 32 years, with HIV/AID, PCOS, infertility, and PMS being the primary contributors to the increase. Furthermore, women aged 15–24 years exhibited the most significant increase in CGD burdens. The burden of CGDs is not evenly distributed, and lower SDI countries have still shown higher burdens. According to the BAPC model, by 2031, the global ASDR and ASMR of CGDs may show a downward trend, whereas the ASIR and ASPR in upward trajectory. Thus, we hope that this study can provide detailed information for policymakers to allocate medical resources, implement targeted and effective measures, and facilitate collaboration between regions to improve gynecological diseases care in lower-income countries. To further address the burdens, future research should consider the inclusion of data on risk factors, evaluating targeted interventions, and strengthening health system responses related to gynecologic diseases to provide a more comprehensive assessment.

## Supplementary Information

Additional file 1.

Additional file 2.

Additional file 3.

Additional file 4.

Additional file 5.

Additional file 6.

Additional file 7.

Additional file 8.

Additional file 9.

Additional file 10.

Additional file 11.

Additional file 12.

Additional file 13.

Additional file 14.

Additional file 15.

Additional file 16.

Additional file 17.

Additional file 18.

Additional file 19.

## Data Availability

The data for this study are available from Global Burden of Disease Study 2021 at https://ghdx.healthdata.org/gbd-2021
