# Supplementary material for: Rising global burden of common gynecological diseases in women of childbearing age from 1990 to 2021: an update from the Global Burden of Disease Study 2021
Source: Reprod Health. 2025 Apr 21;22:57. doi: 10.1186/s12978-025-02013-1 (PMC12010537; doi:10.1186/s12978-025-02013-1)
Supplement: Supplementary file 2 — Additional file 2. [file 12978_2025_2013_MOESM2_ESM.docx]

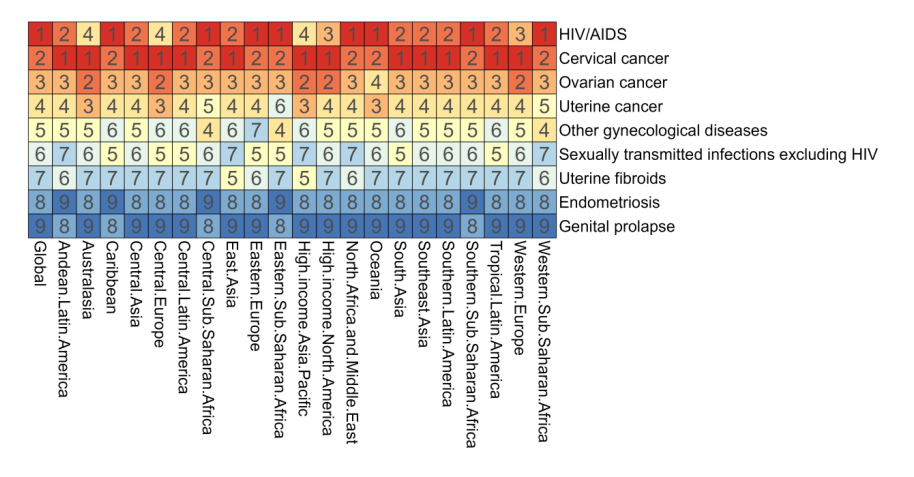
Figure S1. Ranking of age-standardized mortality rates for different types of gynecological disorders among women of childbearing age by GBD region in 2021.


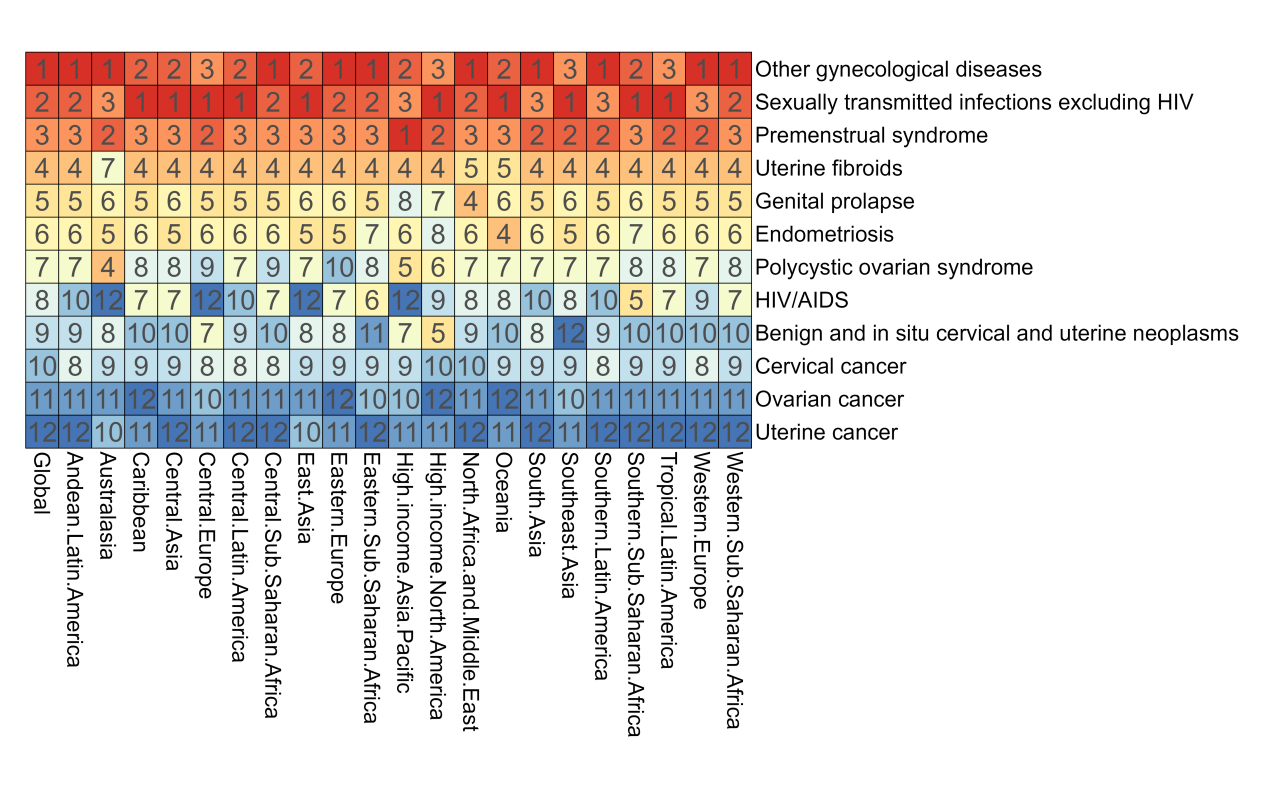


Figure S2. Ranking of age-standardized incidence rates for different types of gynecological disorders among women of childbearing age by GBD region in 2021.


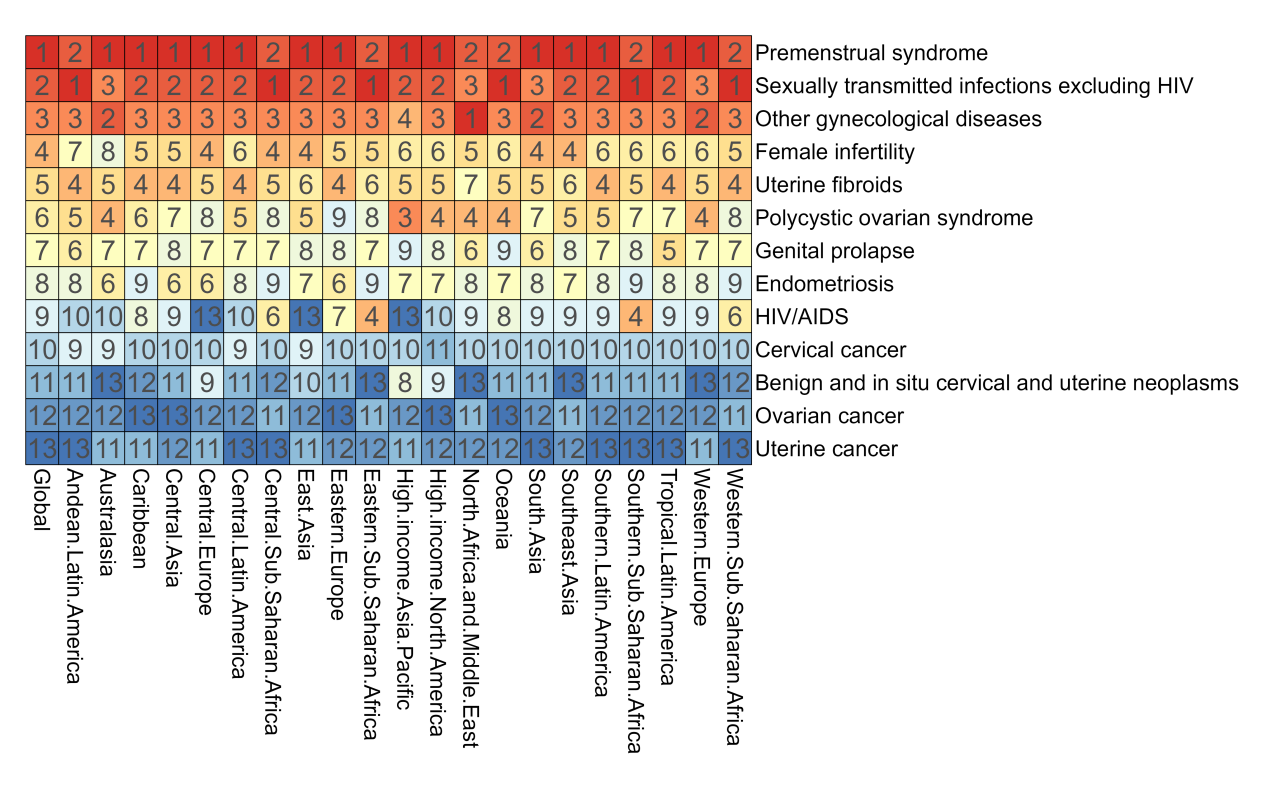


Figure S3. Ranking of age-standardized prevalence for different types of gynecological disorders among women of childbearing age by GBD region in 2021.


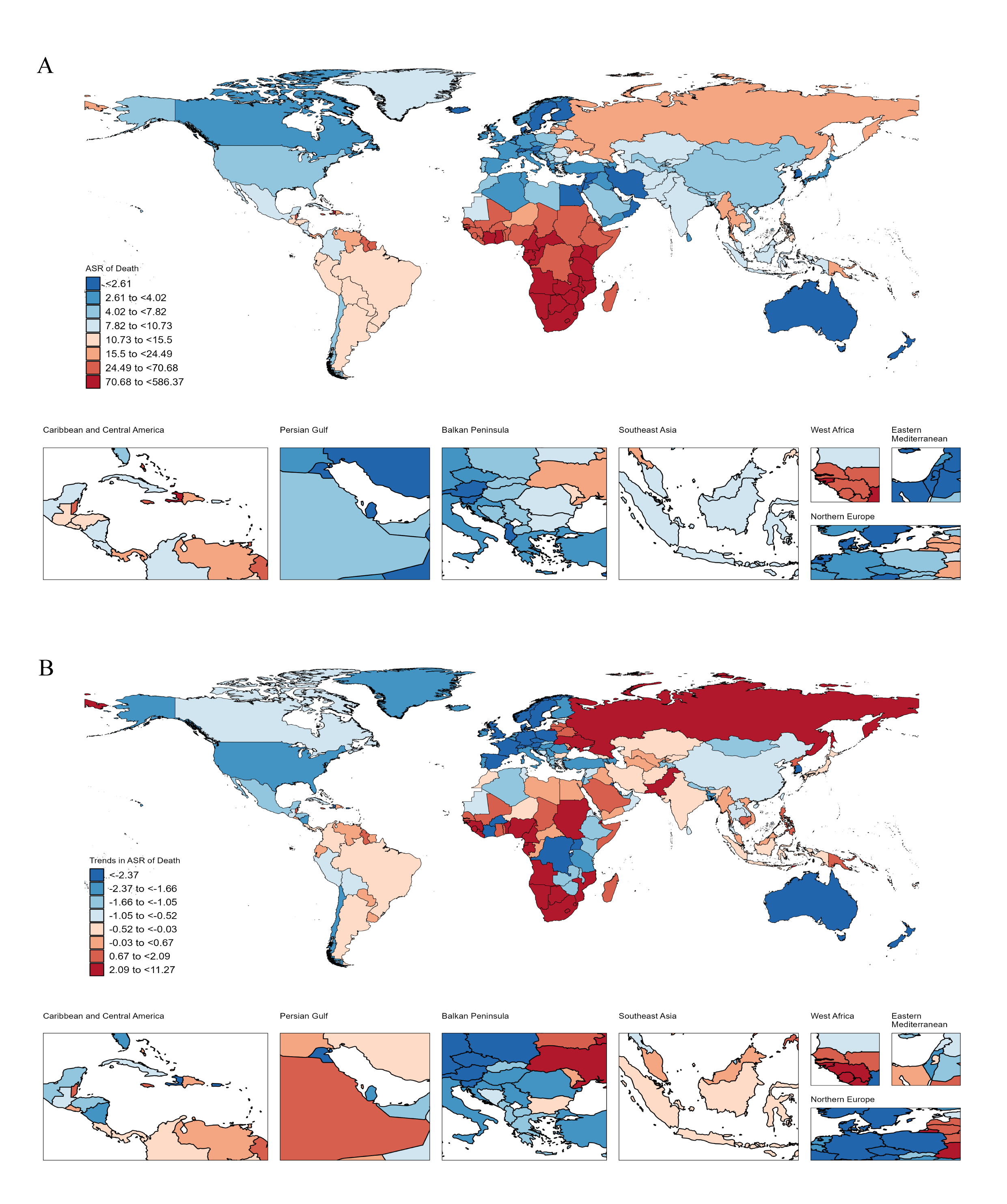


Figure S4. National age-standardized death rates of combined gynecological disorders (CGDs) among women of childbearing age in 2021 and their average annual percentage change from 1990 to 2021. (A) Age-standardized death rates; (B) AAPC for age-standardized death rates. Abbreviations: AAPC, average annual percentage change. Note: Map lines delineate study areas and do not necessarily depict accepted national boundaries, which however was not the key point for this study.


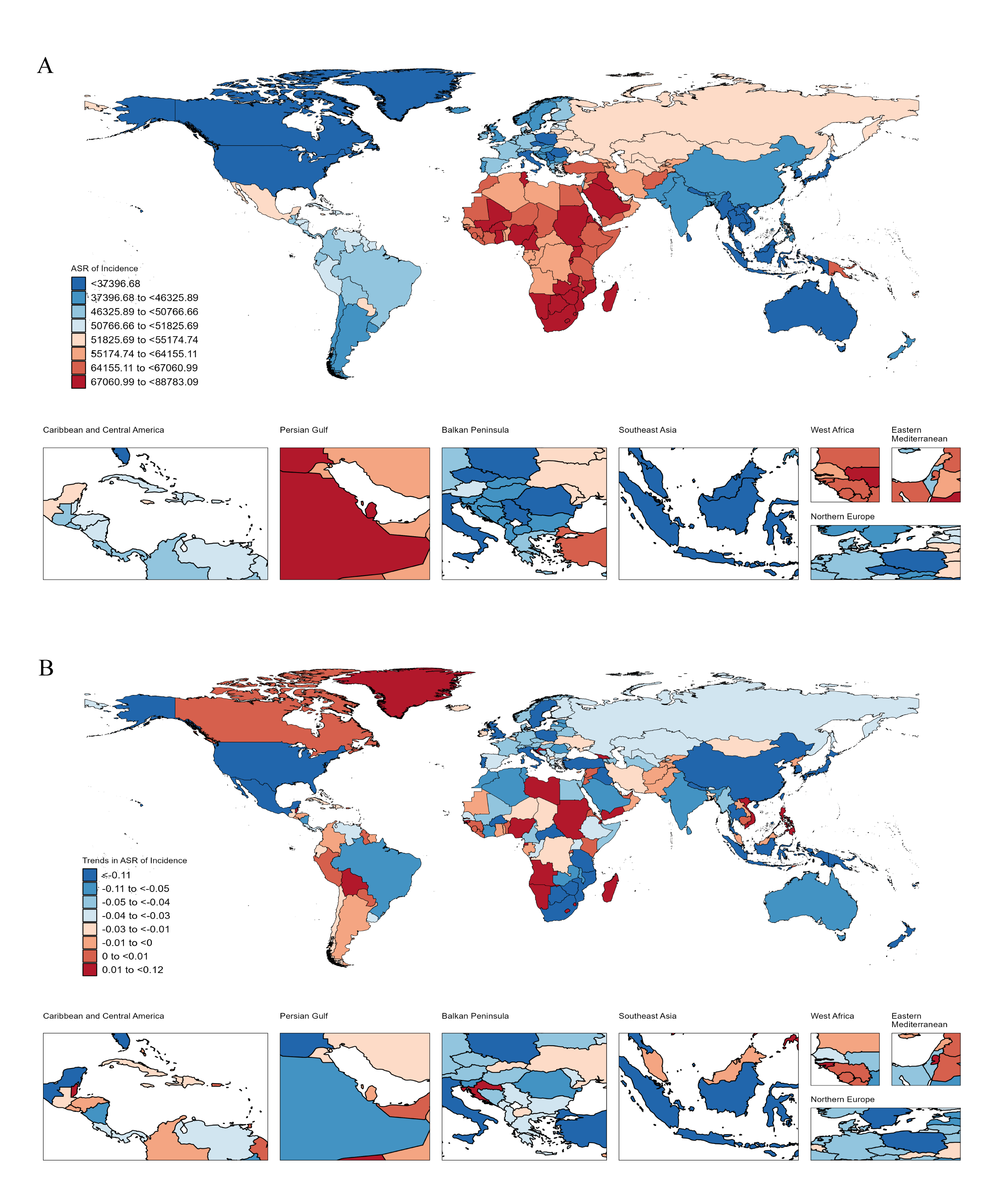


Figure S5. National age-standardized incidence rates of combined gynecological disorders (CGDs) among women of childbearing age in 2021 and their average annual percentage change from 1990 to 2021. (A) Age-standardized incidence rates; (B) AAPC for age-standardized incidence rates. Abbreviations: AAPC, average annual percentage change. Note: Map lines delineate study areas and do not necessarily depict accepted national boundaries, which however was not the key point for this study.


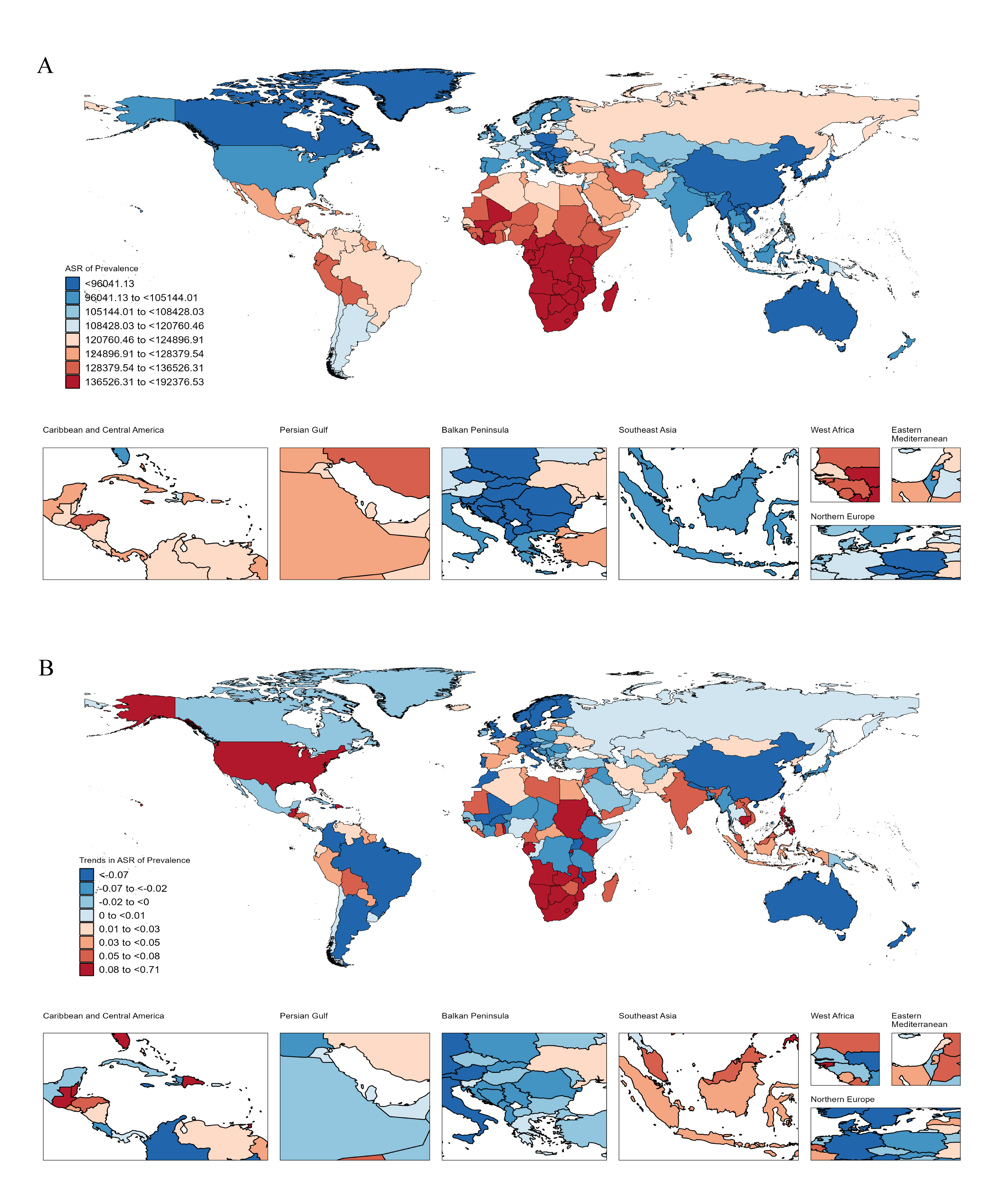


Figure S6. National age-standardized prevalence rates of combined gynecological disorders (CGDs) among women of childbearing age in 2021 and their average annual percentage change from 1990 to 2021. (A) Age-standardized prevalence rates; (B) AAPC for age-standardized prevalence rates. Abbreviations: AAPC, average annual percentage change. Note: Map lines delineate study areas and do not necessarily depict accepted national boundaries, which however was not the key point for this study.


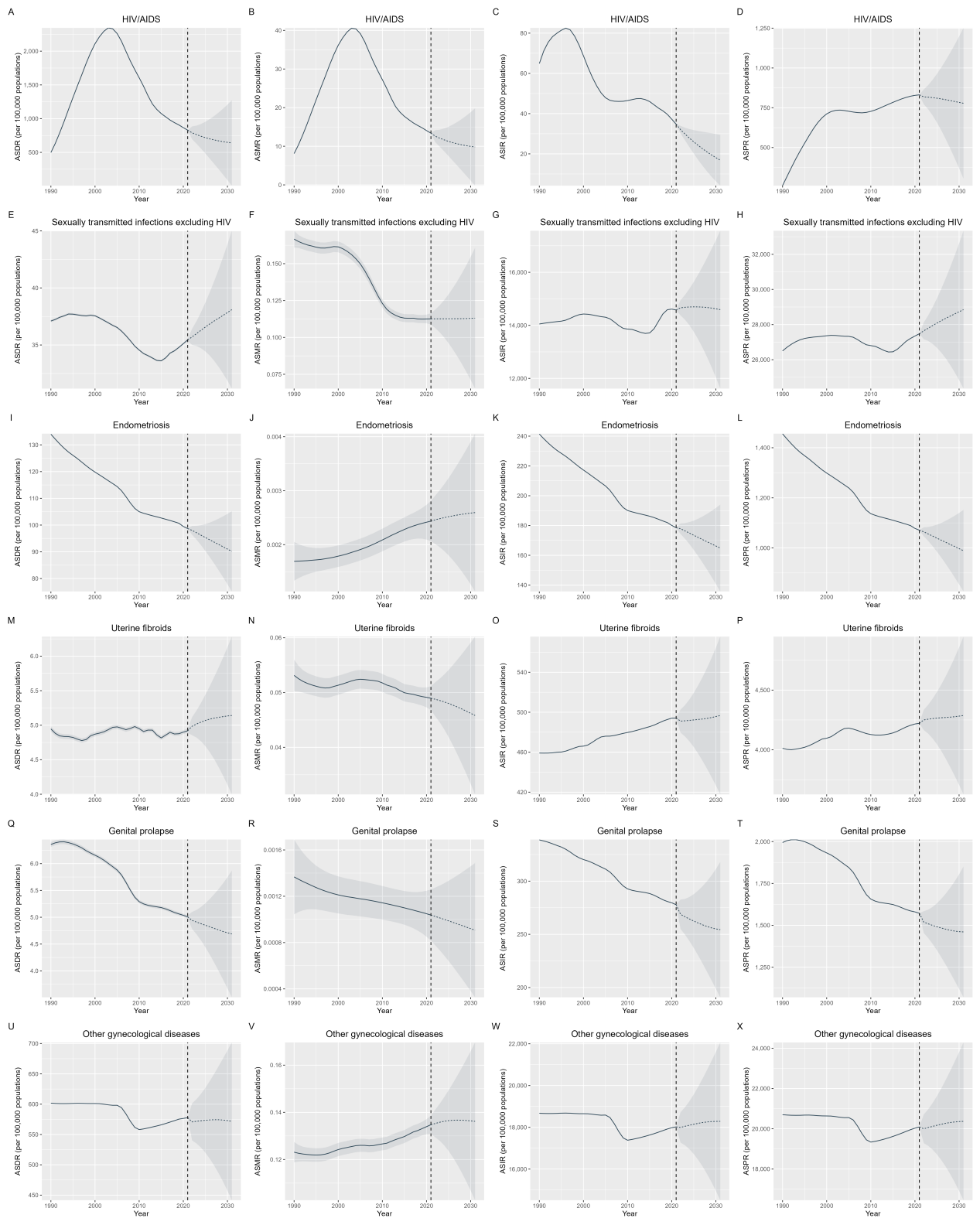


Figure S7. Projections of different types of gynecological diseases burden in women of childbearing age by 2031 based on the Bayesian age-period-cohort (BAPC) models. (A-D) HIV/AIDS; (E-H) Sexually transmitted infections excluding HIV; (I-L) Endometriosis; (M-P) Uterine fibroids; (Q-T) Genital prolapse; (U-X) Other gynecological diseases. Abbreviations: DALYs disability-adjusted life-years; ASDR age-standardized DALYs rate; ASMR age-standardized mortality rate；ASIR age-standardized incidence rate；ASPR age-standardized prevalence rate.


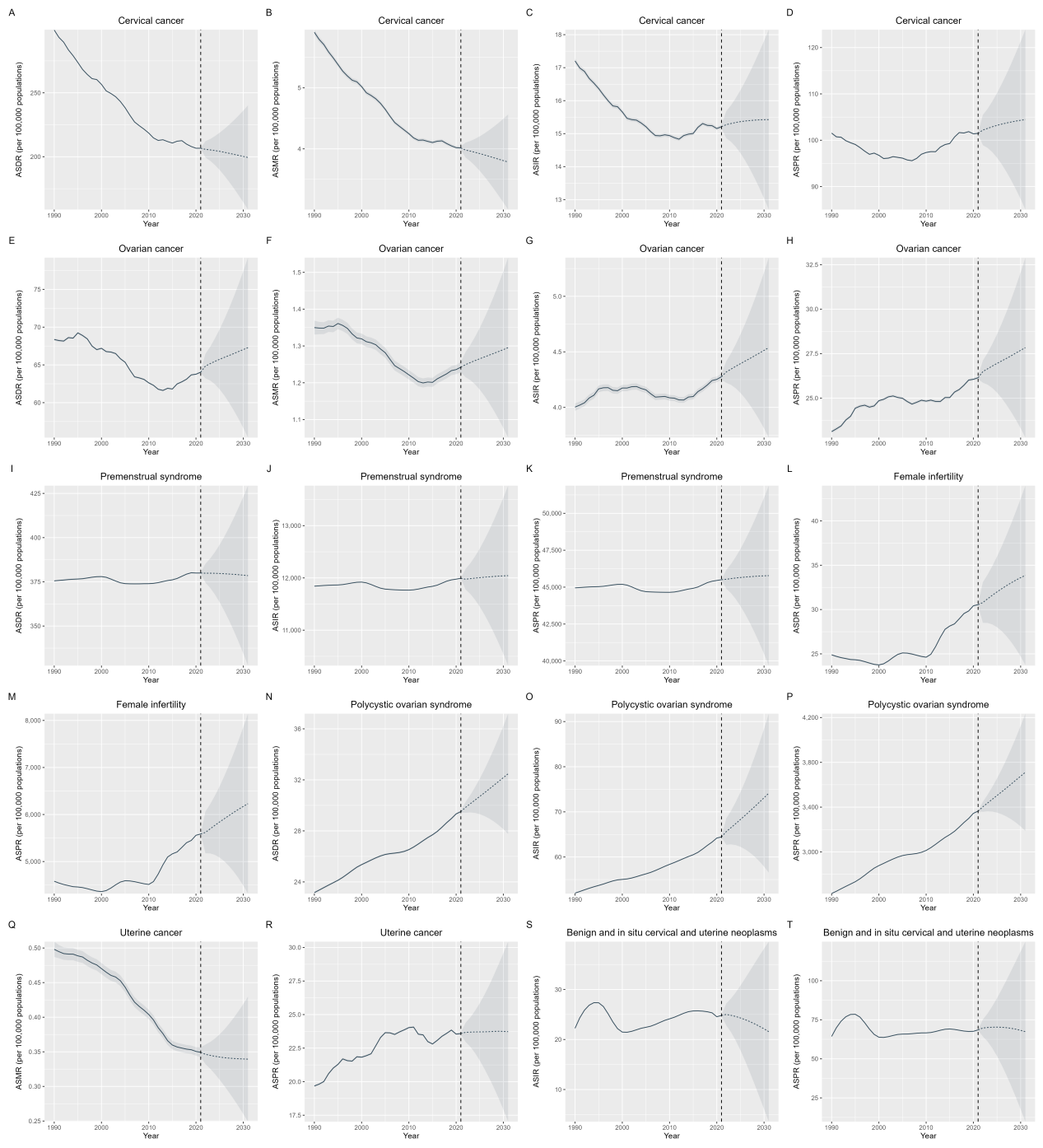


Figure S8. Projections of different types of gynecological diseases burden in women of childbearing age by 2031 based on the Bayesian age–period–cohort (BAPC) models (continued). (A-D) Cervical cancer; (E-H) Ovarian cancer; (I-K) Premenstrual syndrome; (L-M) Female infertility; (N-P) Polycystic ovarian syndrome; (Q-R) Uterine cancer; (S-T) Benign and in situ cervical and uterine neoplasms. Abbreviations: DALYs disability-adjusted life-years; ASDR age-standardized DALYs rate; ASMR age-standardized mortality rate；ASIR age-standardized incidence rate；ASPR age-standardized prevalence rate.
